# Are Furanocoumarins Present in the Cichorieae Tribe of Asteraceae? A Comparative Study of *Cicerbita alpina* (Asteraceae) and *Peucedanum ostruthium* (Apiaceae)

**DOI:** 10.3390/plants14182815

**Published:** 2025-09-09

**Authors:** Calisto Moreno Cardenas, Gaia Maria Francesca Grieco, Dimitrina Zheleva-Dimitrova, Giovanni Appendino, Christian Zidorn

**Affiliations:** 1 Pharmazeutisches Institut, Abteilung Pharmazeutische Biologie, Christian-Albrechts-Universität zu Kiel, 24118 Kiel, Germany; ccardenas@pharmazie.uni-kiel.de; 2 Department of Pharmacy, School of Medicine and Surgery, University of Naples Federico II, 80131 Naples, Italy; gaia.grieco@studenti.unina.it; 3 Department of Pharmacognosy, Faculty of Pharmacy, Medical University-Sofia, 1000 Sofia, Bulgaria; dzheleva@pharmfac.mu-sofia.bg; 4 Dipartimento di Scienze del Farmaco, Università del Piamonte Orientale, 28100 Novara, Italy; giovanni.appendino@uniupo.it; 5 Division of Pharmaceutical Biotechnology, Department of Pharmaceutical Biology and Biotechnology, Wroclaw Medical University, Borowska 211, 50-556 Wrocław, Poland

**Keywords:** *Cicerbita alpina*, *Peucedanum ostruthium*, chemophenetics, furanocoumarins

## Abstract

*Cicerbita alpina* (L.) Wallroth and *Peucedanum ostruthium* W.D.J. Koch occur in megaphorb communities in alpine and subalpine areas; both species often share the same habitats. *P. ostruthium* is used as a spice for spirits, while young shoots of *C. alpina* are collected in the northeastern regions of Italy as a local delicacy. In the present study, we isolated eleven known coumarins and one chromone from subaerial parts of *P. ostruthium*; two furanocoumarins were found for the first time in this species. Using UHPLC-HRMS, we analyzed the furanocoumarin content of two *P. ostruthium* accessions, one commercially purchased and one collected in the wild. These samples were compared to six rootstock samples of *Cicerbita alpina* collected in the wild. Though the furanocoumarins imperatorin, isoimperatorin, oxypeucedanin, and ostruthol had been reported from *C. alpina* before, we were not able to detect any of these compounds in our samples of *C. alpina*. Therefore, and due to the occurrence of both taxa in the same habitat, we assume that the original report of furanocoumarins in *C. alpina* was based on a mixed collection of *C. alpina* and *P. ostruthium*. This hypothesis seems plausible, because furanocoumarins have not been reported from any other taxon of the Cichorieae tribe of the Asteraceae family.

## 1. Introduction

Furanocoumarins are a class of specialized natural products occurring in some families of higher plants, which are, however, not closely related. Furanocoumarins are classified into linear and angular furanocoumarins, based on the angle between furane ring and coumarin moiety. Furanocoumarins are found *inter alia*, in the Apiaceae, Fabaceae, Moraceae, and Rutaceae families. Furanocoumarins serve important ecological functions for the taxa producing them, e.g., as chemical defenses against herbivores and pathogens, but also as phytoalexins in response to biotic stress [1,2,3].

*Peucedanum ostruthium* W.D.J. Koch (vernacular name “masterwort”, German “Meisterwurz”) is a perennial herb native to the mountainous regions of Europe. The taxon has traditionally been used in the preparation of liqueurs, bitters, and herbal teas [4,5,6,7]. *Peucedanum ostruthium*, and the genus *Peucedanum* in general, are known to contain furanocoumarins. The genus *Peucedanum* comprises approximately 120 species and furanocoumarins have been detected in many of them [8].

*Cicerbita alpina* (L.) Wallroth is a member of the Cichorieae tribe of the Asteraceae family. *C. alpina* is, like *P. ostruthium*, distributed in the mountainous regions of Europe. It is locally used as a delicacy in some alpine regions of Italy [9]. Sesquiterpene lactones and in particular sesquiterpene lacone glucosides are the most characteristic compounds found in *C. alpina*. Sesquiterpene glucosides are excellent chemophenetic markers within and for the Cichorieae tribe of the Asteraceae family [10]. Compounds identified in the roots of *C. alpina* include bitter tasting lactucin derivatives and sonchusides [11,12]. Phytochemical analysis of extracts of young shoots of *C. alpina* revealed a diverse array of phenolic constituents, exhibiting both significant antioxidant activity and anthelmintic effects [13,14,15]. *C. alpina* is currently the only species within the Cichorioieae tribe of the Asteraceae family for which furanocoumarins have been reported [13,16]. Furanocoumarins have previously been reported only for a few species of the Asteraceae, belonging to the subfamily Mutisioideae as well as in “*Artemisia reticulata*”. However, the latter report is hard to assess, as the name “*Artemisia reticulata*” seems never to have been validly published and does not appear in any of the major taxonomic online databases [17,18].

As *Cicerbita alpina* sprouts are locally eaten as a vegetable collected from the wild in parts of Northern Italy, we aimed to determine whether furanocoumarins actually occur in *C. alpina*, or whether the previous report was based on mixed collection of *C. alpina* with *P. ostruthium*. Roots of *P. ostruthium* are traditionally used in medicine and known to contain high amounts of furanocoumarins. Since furanocoumarins are pharmacologically active, but may also pose potential health risks, clarifying their presence in *C. alpina* is of considerable interest to guarantee the safety of its traditional dietary use. This regards in particular the known phototoxicty of furanocoumarins.

**Figure 1:**
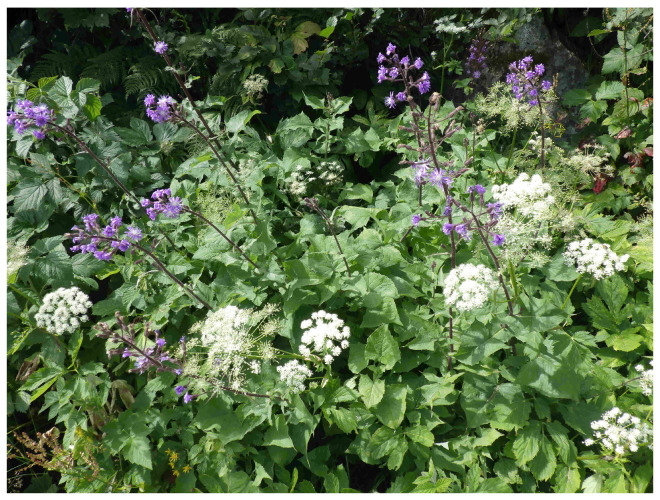
*Cicerbita alpina* and *Peucedanum ostruthium* growing together in a subalpine megaphorb community (5th of August 2024 near Passo del Tonale, Vermiglio/Trentino/Italy; N 46°15′12.0″, E 10°36′18.5″ E, 2000 m. a.m.s.l.; CZ).

*C. alpina* and *P. ostruthium* frequently co-occur (Figure 1) in parts of their distribution range, in particular in the Alps. We therefore hypothesized that the furanocoumarins previously reported from roots of *C. alpina* in fact originated from a mixed collection of *C. alpina* and *P. ostruthium*. Roots of *P. ostruthium* are known to contain high amounts of furanocoumarins. To support our hypothesis, we isolated twelve coumarins from *P. ostruthium* and carried out a comparative UHPLC-HRMS analysis of acetone extracts from six accessions of *C. alpina* and two samples of *P. ostruthium*.

## 2. Results

### 2.1. Isolation and Identification

**Figure 2:**
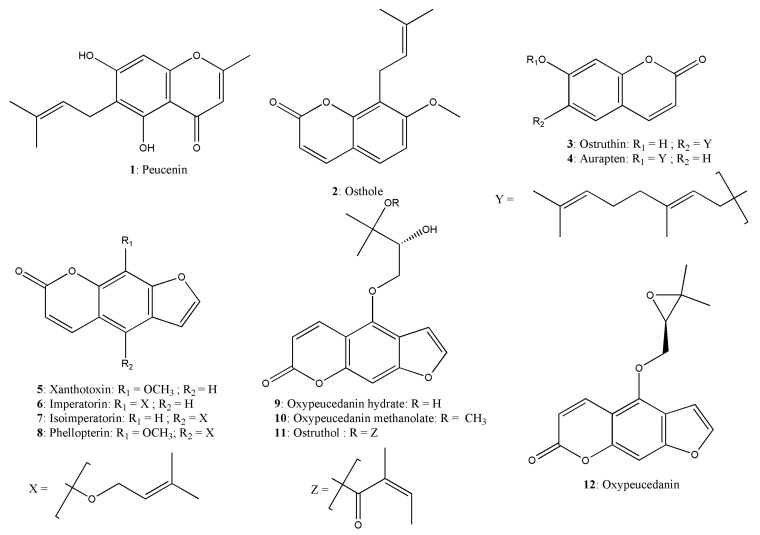
Chemical structures of isolated compounds from *P. ostruthium*.

Exhaustive maceration, followed by repeated chromatographic separation led to the isolation of one prenylated chromone (**1**), three prenylated coumarins (**2** to **4**) and eight linear furanocoumarins (**5** to **12**) (Figure 2). After comparison of mass spectrometry (MS) and nuclear magnetic resonance (NMR) data, these compounds were identified as peucenin (**1**) [19], osthole (**2**) [20], ostruthin (**3**) [21], auraptene (**4**) [22], xanthotoxin (**5**) [23], imperatorin (**6**) [24], isoimperatorin (**7**) [25], phellopterin (**8**) [26], oxypeucedanin hydrate (**9**) [27], oxypeucedanin methanolate (**10**) [28], ostruthol (**11**) [29], and oxypeucedanin (**12**) [30]. To the best of our knowledge, phellopterin (**8**) and xanthotoxin (**5**) are reported here for the first time from *P. ostruthium*. NMR and MS data for these compounds are provided in the Supplementary Material.

### 2.2. Comparative HPLC Analysis

**Figure 3:**
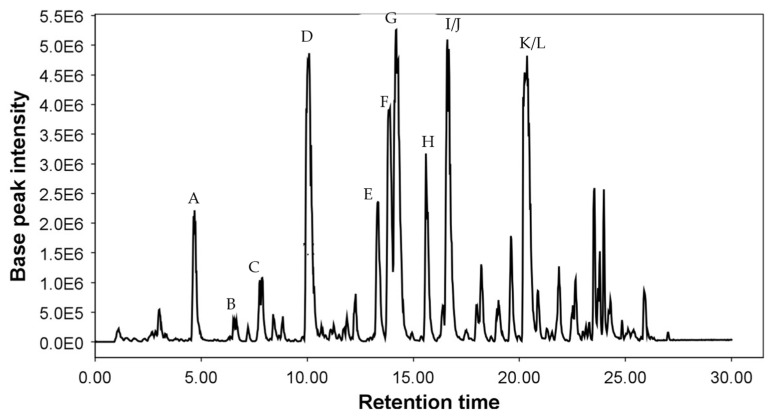
Base peak chromatogram of *P. ostruthium* acetone extract. Peak A = oxypeucedanin hydrate (**9**), B = xanthotoxin (**5**), C = oxypeucedanin methanolate (**10**), D = oxypeucedanin (**12**), E = peucenin (**1**), F = ostruthol (**11**), G = imperatorin (**6**), H = phellopterin (**8**), I = isoimperatorin (**7**), J = osthole (**2**), K = ostruthin (**3**), L = auraptene (**4**).

Acetone extracts from six accessions of *Cicerbita alpina* were analyzed and compared with those from two samples of *Peucedanum ostruthium* using a modified version of the chromatographic method of Vogl et al. [4]. In the base peak chromatogram of the commercial sample of *Peucedanum ostruthium* (P2) shown in Figure 3, all previously isolated furanocoumarins were detected. The corresponding high-resolution accurate mass spectrometric data for these compounds are summarized in Table 1.

**Figure 4:**
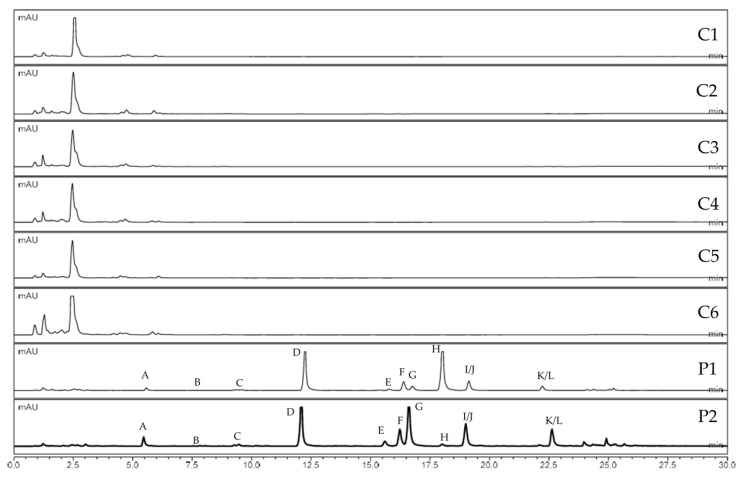
Comparison of UV chromatograms (254 nm) of acetone extracts from *C. alpina* accessions (C1–C6) and *P. ostruthium* samples (P1 and P2).

UHPLC-HRMS data sets were processed using MZmine, applying a noise threshold of 5.0 × 10^2^ [31]. All isolated compounds were detected in both *P. ostruthium* samples (P1 and P2). In contrast, none of the furanocoumarins, including the ones previously described from *C. alpina*, were detected in any of the six samples of *C. alpina* (C1–C6). Figure 4 indicates the absence of apolar compounds, including furanocoumarins, in the extracts of *C. alpina*.

Interestingly, the sample of *Peucedanum ostruthium* collected in the wild, contained higher amounts of phellopterin (**8**), but markedly lower amounts of isoimperatorin (**9**) than the commercial sample.

## 3. Discussion

Furanocoumarins were not detectable in rootstocks of *Cicerbita alpina*. This fact conforms with existing chemophenetic data for other members of the Cichorieae tribe of the Asteraceae family. In fact, the discrepancy between the reported occurrence of furanocoumarins in *C. alpina* and the lack of reports of furanocoumarins from any other member of the Cichorieae tribe was one of the main motivations for this study. Within the Asteraceae family, the subfamily Mutisioideae has been reported to contain furanocoumarins. However, this subfamily is phylogenetically quite distant from the Cichorieae [18,32,33]. The detection of ostruthol (**11**) by Zheleva-Dimitrova et al. through LC-MS/MS analysis underscores the critical role of reference standards in the accurate identification of natural products [13].

The assignment of compound structures based solely on MS/MS spectral comparison, presents significant perils for erroneous positive reports (in particular, when “only” confirming previous—in this case erroneous—studies. Zheleva-Dimitrova et al. [13] adopted the identification confidence framework proposed by Schymanski et al., which ranks compound annotations by confidence levels—Level 1 indicating confirmation via comparison with an authentic reference standard, and Level 2 representing a probable structure based on spectral library matching [34]. Çiçek et al. proposed a new ranking system of confidence levels for the identification of phytochemical metabolites [35]. According to this classification system, only NMR data combined with an additional technique providing comprehensive structural information—such as high-resolution mass spectrometry (HR-MS) or X-ray crystallography—is considered sufficient to achieve the highest confidence level in structural annotation (Level A). In contrast, the sole reliance on MS data from spectral libraries, without the support of authentic standards or retention indices, is deemed inadequate for confirming the presence of natural products with a very high confidence level (Level C). False positive reports based on LC-MS data are a growing problem in phytochemical analysis. Inaccurate compound identification easily leads to the erroneous re-reporting of the same compound in subsequent studies, based to the expected presence. Another persistent challenge in comparative natural product chemistry is the frequent lack of adequate control materials, including the proper storage of voucher specimens in recognized institutions (and not simply in laboratory collections which, due to relocation/retirement of their reference person cannot guarantee constant availability). This issue is particularly pronounced for studies published in the last century, for which verification of the original samples is often no longer feasible. As a result, inaccurate reports may be accepted as valid and perpetuated in subsequent publications, thereby perpetuating the issue and making retrospective corrections increasingly challenging. In cases of sample contamination with material from a different species, accurate and detailed information regarding the collection site, the taxonomic identification procedures, and associated plant species is essential to determine whether the sample may represent a mixture of two species [36]. However, apart from exercising due diligence during plant collection and ideally taking pictures of the collection site during collection, this kind of error seems to be particularly hard to avoid and even more difficult to detect in retrospect.

The occurrence of furanocoumarins has been extensively investigated in the Apiaceae family, with significant progress made in elucidating their biosynthetic pathways and the underlying genetic mechanisms [37,38,39,40,41]. This study constitutes the first report of xanthotoxin (**5**) and phellopterin (**8**) in *Peucedanum ostruthium*. Within the genus *Peucedanum*, xanthotoxin (**5**) has previously been identified in *P. formosanum* Hayata, *P. galbanum* Benth. & Hook.f., *P. grande* C.B. Clarke, *P. harry-smithii* var. *subglabrum* (R.H. Shan & Sheh) R.H. Shan & M.G. Sheh, *P. japonicum* Thunb., *P. luxurians* Tamamsch., *P. palustre* (L.) Moench, and *P. praeruptorum* Dunn [42,43,44,45,46,47,48]. Phellopterin **8** has been reported from *P. medicum* var. *gracile* Dunn ex R.H. Shan & M.L. Sheh *P. palustre* (L.) Moench, and *P. zenkeri* Engl. [45,49,50]. Within the genus *Peucedanum*, the presence of furanocoumarins is a well-documented chemophenetic trait.

In general, the presence or absence of particular secondary metabolite may also be related to environmental or developmental factors, which all influence the array of compounds contained in an individual plant at a specific point in time. Also, the isolation procedure significantly affects the array of compounds detectable in an extract. Therefore, it is essential to include accessions from multiple regions and to apply identical sample preparation methods in comparative phytochemical studies.

In conclusion, furanocoumarins were absent in all six accessions of *C. alpina* investigated. Given the frequent co-occurrence of *C. alpina* and *P. ostruthium*, the previous report of furanocoumarins by Appendino et al. [16] is most easily explained by a mixed collection of these two species. The subsequent detection of ostruthol **11** by UHPLC-HRMS in aerial parts and flowering heads of *C. alpina* from Bulgaria with a confidence level of 2 at the Schimansky scale [34], is probably a false positive report, induced by the fact that ostruthol **11** had already been reported from *C. alpina* and was thus also to be expected in the analyzed extracts.

## 4. Materials and Methods

### 4.1. Plant Material, Reagents and Experimental Procedures

Two kilograms of dried root stock of *Peucedanum ostruthium* (L.) W.D.J.Koch were purchased from Alfred Galke GmbH, Am Bahnhof 1, 37539 Bad Grund, Germany. Three accessions of *Cicerbita alpina* (L.) Wallr. were collected in May 2025 from the Harz Mountains, Lower Saxonia, Germany. The collection sites were as follows: C1, south of Altenau/Goslar/Lower Saxonia/Germany, N 51°47′20.4″, E 10°26′54.6″, 586 m a.m.s.l. (CMC-202504524-3), C2, north of Clausthal-Zellerfeld/Goslar/Lower Saxonia/Germany, N 51°50′05.4″, E 10°20′26.4″, 529 m a.m.s.l. (CMC-20250524-2), and C3, northwest of Braunlage/Goslar/Lower Saxonia/Germany, N 51°44′29.1″, E 10°36′21.2″, 637 m a.m.s.l. (CMC-20250524-6). C4 was collected in May 2025 W Žacléř/Trutnov/Hradec Kralove/Czech Republic, N 50°39′57.0″, E 15°51′52.7″, 950 m a.m.s.l. (CZ-20250531B-1). C5 was collected E Karpacz Górny/Jelenia Góra/Dolnoślaskie/Poland, N 50°46′12.4″, E 15°43′50.9″, 790 m a.m.s.l. (CZ-20250529B-1). C6 was collected in Vitosha Mountains/Sofia/Oblast Sofia/Bulgaria, near Hotel Moreni, N 42°35′28.7″, E 23°17′35.0″, 1780 m. a.m.s.l. Voucher specimens are deposited under the herbarium of Kiel University (KIEL0005021-KIEL0005026) and the Bulgarian Academy of Sciences (SOM179541). P1 was collected S Karpacz Górny/Jelenia Góra/Dolnoślaskie/Poland, N 50°46′53.6″, E 15°43′19.4″, 860 m a.m.s.l. (CZ-20250529A-1).

LC-MS-grade acetonitrile and water, as well as other (analytical grade) solvents and reagents, were purchased from VWR International GmbH (Darmstadt, Germany). LC-MS-grade formic acid was purchased from Sigma–Aldrich Co. (St. Louis, MO, USA). The water used in the semi-preparative HPLC was double-distilled in-house. LC-MS Grade formic acid was purchased from Sigma-Aldrich Co. (St. Louis, MO, USA). TLC was performed on silica gel 60 F254 plates (VWR International, Darmstadt, Germany) using Dichlormethane and Petrolether (1:1) as the mobile phase and 366 nm UV-Light for detection. Column chromatography was performed using a Sephadex LH-20 column (GE Healthcare AB, Uppsala, Sweden) and Silica gel 60 (Carl Roth GmbH + Co. KG, Karlsruhe, Germany). Semi-preparative HPLC was performed using an Ultimate 3000 instrument equipped with an HPG-3400SD pump, WPS-3000SL autosampler, TCC-3000SD column heater, and VWD-3400RS variable wavelength detector (Thermo Fisher Scientific Inc., Waltham, MA, USA) using a Phenomenex Aqua column (5 µm, 250 × 10 mm). Extracts, fractions and pure compounds were analyzed on a Shimadzu Nexera 2 liquid chromatograph connected to a LC-MS triple quadrupole mass spectrometer with electrospray ionization (Shimadzu, Kyoto, Japan). The comparative HPLC-MS analysis was performed using a Shimadzu Nexaera 4 liquid chromatograph connected to a SciEx X500r qTOF (AB Sciex LLC, Marlborough, MA, USA). For separation a Phenomenex Luna Omega polar C18 column (100 × 2.1 mm, 1.6 µm particle size, Phenomenex, Aschaffenburg, Germany) was used. 1D (^1^H and ^13^C) and 2D (HSQC, HMBC, and COSY) NMR spectra were recorded on a Bruker Avance III 400 NMR spectrometer operating at 400 MHz for the proton channel and 100 MHZ for the ^13^C channel with a 5 mm PABBO broadband probe with a z-gradient unit at 298 K (Bruker BioSpin GmbH, Rheinstetten, Germany). Reference Values were 7.26 (^1^H) and 77.16 (^13^C) for Chloroform. Structure elucidation was performed on Topspin 3.6 software (Bruker Biospin GmbH, Rheinstetten, Germany). 5 mm NMR sample tubes were obtained from Rototec-Spintec GmbH, Griesheim, Germany.

### 4.2. Extraction and Isolation

Powdered roots of *P. ostruthium* (1.00 kg) were extracted by maceration, after sonification for 15 min, at room temperature for two weeks. The solvents used were acetone (10.5 L) for 7 days, followed by methanol–acetone–water (3:1:1) (10.5 L) for additional seven days, yielding 112.9 g and 167.1 g of crude extract, respectively. The acetone extract (112.9 g) was further processed by vacuum liquid chromatography (VLC), using a stepwise elution with 800 mL of hexane, followed by 500 mL each of increasing mixtures of hexane/dichloromethane (1, 2, 5, 25, 50 and 100%), then dichloromethane/methanol (1, 2, 5, 25, 50 and 100%) yielding thirteen fractions (1–13). The first three fractions were combined (Fraction A, 20.3 g) and subjected to further separation by silica gel column chromatography (ID 50 mm, 205 g of silica) using the same gradient solvent system as previously described yielding 80 fractions (A1–A80) of which fraction A52 consisted of 1.00 g of **7** and the methanol insoluble part of A54 126 mg of **6**. Sephadex LH-20 column chromatography of fraction A49 afforded eleven subfractions (A49A–A49K). Subfraction A49F was further purified by semi-preparative HPLC, yielding the isolation of **2** (7 mg), **4** (32 mg) and **9** (6 mg). Similarly, fractions A55 and A62 were subjected to semi-preparative HPLC, resulting in the isolation of **1** (1 mg), **3** (3 mg), **5** (2 mg), **8** (6 mg), **10** (2 mg), **12** (2 mg) and **11** (2 mg).

### 4.3. Sample Preparation for HPLC Analysis

A total of 0.500 g of dried plant material from each accession of *C. alpina* and *P. ostruthium* was extracted three times for 15 min each using 10 mL of acetone in an ultrasonic bath. The combined extracts were evaporated to dryness under reduced pressure using a Büchi rotavapor R300 and re-dissolved in 1.5 mL of methanol for further analysis.

### 4.4. HPLC Analysis

HPLC-MS analyses were performed using a modified gradient based on the method described by Vogl et al. [4], employing 0.1% formic acid in water as solvent A and 0.1% formic acid in acetonitrile as solvent B. The gradient profile was as follows: 25% B at 0 min; a linear increase to 37% B from 0 to 5.72 min; 45% B from 5.72 to 12.74 min; 65% B from 12.74 to 21.51 min; 95% B from 21.51 to 22.39 min; and maintained at 95% B until 30 min. The chromatographic separation was performed at a flow rate of 0.300 mL/min. The column oven temperature was maintained at 38 °C. Detection was carried out using a UV detector (254 nm) and a quadrupole time-of-flight mass spectrometer (qTOF-MS) operated in positive ion mode, with a mass range of *m*/*z* 100–1000.

## Figures and Tables

**Table 1:** List of compounds **1**–**12** and their high-resolution accurate mass spectrometric data in *P. ostruthium*.

Retention Time [min]	Peak	Nr.	Common Name	M	[M+H]^+^ (*m*/*z*)	Error (ppm)	Molecular Formula	Major HRMS^2^ Fragments (*m*/*z*)	UV Maxima
13.64	E	**1**	Peucenin	260	261.1109	−6.83	C_15_H_16_O_4_	205.0492, 165.0175, 123.0069	223, 251, 297
16.79	J *	**2**	Osthole	244	245.1154	−9.67	C_15_H_16_O_3_	131.0490, 103.0544, 159.0438	204, 256, 320
20.50	K *	**3**	Ostruthin	298	299.1614	−11.10	C_19_H_22_O_3_	147.0431, 176.0462, 187.0386	203, 331
20.50	L *	**4**	Auraptene	298	299.1621	−8.76	C_19_H_22_O_3_	119.0482, 163.0388, 81.0694	206, 321
6.78	B	**5**	Xanthotoxin	216	217.0490	−4.99	C_12_H_8_O_4_	174.0300, 202.0235, 161.0577	220, 245, 303
14.58	G	**6**	Imperatorin	270	271.0959	−4.18	C_16_H_14_O_4_	147.0434, 203.0338, 175.0383	218, 249, 304
16.79	I *	**7**	Isoimperatorin	270	271.0957	−4.92	C_16_H_14_O_4_	147.0433, 131.0490, 203.0337	221, 254, 312
15.99	H	**8**	Phellopterin	300	301.1057	−6.31	C_17_H_16_O_5_	218.0197, 217.0116, 162.0310	222, 270, 315
4.87	A	**9**	Oxypeucedanin hydrate	304	305.0995	−9.88	C_16_H_16_O_6_	203.0338, 147.0434, 131.0491	220, 249, 313
8.27	C	**10**	Oxypeucedanin methanolate	318	319.1159	−7.09	C_17_H_18_O_6_	203.0338, 147.0434, 73.0648	221, 253, 312
14.26	F	**11**	Ostruthol	386	387.1418	−6.66	C_21_H_22_O_7_	55.0450, 83.0490, 185.1174	221, 251, 313
10.27	D	**12**	Oxypeucedanin	286	287.0895	−8.53	C_16_H_14_O_5_	147.0430, 203.0333, 59.0488	219, 251, 310

* Compounds I/J and K/L exhibited identical retention times in the LC chromatogram; however, they were successfully separated during the isolation process.

## Data Availability

Original data will be shared on reasonably justified request.

## Supplementary Materials

The following supporting information can be downloaded at: https://www.mdpi.com/article/10.3390/plants14182815/s1.
