# Supplementary material for: Are Furanocoumarins Present in the Cichorieae Tribe of Asteraceae? A Comparative Study of Cicerbita alpina (Asteraceae) and Peucedanum ostruthium (Apiaceae)
Source: Plants (Basel). 2025 Sep 9;14(18):2815. doi: 10.3390/plants14182815 (PMC12473771; doi:10.3390/plants14182815)

Supplementary Material

Are Furanocoumarins Present in the Cichorieae Tribe of Asteraceae? A Comparative Study of *Cicerbita alpina* (Asteraceae) and *Peucedanum ostruthium* (Apiaceae)

Calisto Moreno Cardenas, Gaia Maria Francesca Grieco, Dimitrina Zheleva-Dimitrova, Giovanni Appendino, and Christian Zidorn

1. Chemical shifts of Compound **1** in ppm
2. Chemical shifts of Compound **2** in ppm
3. Chemical shifts of Compound **3** in ppm
4. Chemical shifts of Compound **4** in ppm
5. Chemical shifts of Compound **5** in ppm
6. Chemical shifts of Compound **6** in ppm
7. Chemical shifts of Compound **7** in ppm
8. Chemical shifts of Compound **8** in ppm
9. Chemical shifts of Compound **9** in ppm
10. Chemical shifts of Compound **10** in ppm
11. Chemical shifts of Compound **11** in ppm
12. Chemical shifts of Compound **12** in ppm
13. ^{1}H NMR spectrum of compound **11**
14. ^{13}C NMR spectrum of compound **11**
15. COSY spectrum of compound **11**
16. HSQC spectrum of compound **11**
17. HMBC spectrum of compound **11**
18. MS and MS/MS spectrum of compound **1**
19. MS and MS/MS spectrum of compound **2**
20. MS and MS/MS spectrum of compound **3**
21. MS and MS/MS spectrum of compound **4**
22. MS and MS/MS spectrum of compound **5**
23. MS and MS/MS spectrum of compound **6**
24. MS and MS/MS spectrum of compound **7**
25. MS and MS/MS spectrum of compound **8**
26. MS and MS/MS spectrum of compound **9**
27. MS and MS/MS spectrum of compound **10**
28. MS and MS/MS spectrum of compound **11**
29. MS and MS/MS spectrum of compound **12**

1. Compound **1**, Peucenin, $CDCl_3$, ^{1}H (400 MHz): δ 13.09 (1H, s), 6.33 (1H, s, H-8), 6.01 (1H, bs, H-3), 5.27 (1H, m, *J* = 7.6 Hz), 3.44 (2H, d, *J* = 7.6 Hz), 2.33 (3H, s), 1.84 (3H, s), 1.77 (3H, bs)

2. Compound **2**, Osthole, $CDCl_3$, ^{1}H (400 MHz): δ 7.61 (1H, d, *J* = 9.5 Hz, H-4), 7.28 (1H, d, *J* = 8.6 Hz, H-5), 6.83 (1H, d, *J* = 8.6 Hz, H-6), 6.23 (1H, d, *J* = 9.5 Hz, H-3), 5.22 (1H, tm, *J* = 7.3, 1.4 Hz, H-2′), 3.92 (3H, s, O-CH_3), 3.53 (2H, d, *J* = 7.3 Hz, H-1′), 1.83 (3H, s, H-5′), 1.67 (3H, s, H-4′); ^{13}C (100 MHz): δ 161.5 (C-2), 160.4 (C-7), 153.0 (C-3′), 143.9 (C-4), 132.8 (C-4a), 126.3 (C-5), 121.3 (C-2′), 118.2 (C-8), 113.2 (C-8a), 113.2 (C-3), 107.5 (C-6), 56.2 (O-CH_3), 25.9 (C-5′), 22.1 (C-1′), 18.1 (C-4′)

3. Compound **3**, Ostruthin, $CDCl_{3,,}$ ^{1}H (400 MHz): δ 7.61 (1H, d, *J* = 9.5 Hz, H-4), 7.19 (1H, s, H-5), 6.87 (1H, s, H-8), 6.24 (1H, d, *J* = 9.5 Hz, H-3), 5.31 (1H, td, *J* = 7.2 Hz, H-10), 5.08 (1H, m, H-14), 3.39 (2H, d, *J* = 7.2 Hz, H-9), 2.11 (2H, m, H-13), 2.11 (2H, m, H-12), 1.76 (3H, bs, H-18), 1.69 (3H, bs, H-16), 1.60 (3H, bs, H-17)); ^{13}C (100 MHz): δ 161.9 (C-2), 158.4 (C-7), 154.4 (C-8a), 143.9 (C-4), 139.6 (C-11), 132.2 (C-15), 128.6 (C-5), 125.1 (C-6), 123.9 (C-14), 120.9 (C-10), 113.0 (C-3), 112.6 (C-4a), 103.6 (C-8), 39.8 (C-12), 29.0 (C-9), 26.5 (C-13), 25.9 (C-16), 17.9 (C-17), 16.4 (C-18)

4. Compound **4**, Auraptene, $CDCl_{3,,}$ ^{1}H (400 MHz): δ 7.63 (1H, d, *J* = 9.5 Hz, H-4), 7.35 (1H, d, *J* = 8.5 Hz, H-5), 6.84 (1H, dd, *J* = 8.5 Hz, H-6), 6.82 (1H, m, H-8), 6.24 (1H, d, *J* = 9.5 Hz, H-3), 5.46 (1H, t, *J* = 6.5 Hz, H-10), 5.08 (1H, m, H-14), 4.60 (2H, d, *J* = 6.5 Hz, H-9), 2.12 (2H, m, H-13), 2.10 (2H, m, H-12), 1.76 (3H, s, H-18), 1.66 (3H, s, H-16), 1.60 (3H, s, H-17); ^{13}C (100 MHz): δ 162.3 (C-7), 161.4 (C-2), 156.0 (C-8a), 143.6 (C-4), 142.5 (C-11), 132.1 (C-15), 128.8 (C-5), 123.8 (C-14), 118.6 (C-10), 113.4 (C-6), 113.1 (C-3), 112.6 (C-4a), 101.7 (C-8), 65.6 (C-9), 39.7 (C-12), 26.4 (C-13), 25.8 (C-16), 17.9 (C-17), 16.9 (C-18)

5. Compound **5**, Xanthotoxin, $CDCl_3$, 1H (400 MHz): δ 7.77 (1H, d, *J* = 9.6 Hz, H-4), 7.69 (1H, d, *J* = 2.2 Hz, H-2′), 7.36 (1H, s, H-6), 6.82 (1H, d, *J* = 2.2 Hz, H-3′), 6.38 (1H, d, *J* = 9.6 Hz, H-3), 4.31 (3H, s, O-CH_3)

6. Compound **6**, Imperatorin, $CDCl_3$, ^{1}H (400 MHz): δ 7.76 (1H, d, *J* = 9.6 Hz, H-4), 7.68 (1H, d, *J* = 2.3 Hz, H-2′), 7.35 (1H, s, H-5), 6.81 (1H, d, *J* = 2.3 Hz, H-3′), 6.36 (1H, d, *J* = 9.6 Hz, H-3), 5.61 (1H, tm, *J* = 7.2 Hz, H-2′′), 5.00 (2H, d, *J* = 7.2 Hz, H-1′′), 1.74 (3H, s, H-4′′), 1.71 (3H, s, H-5′′); ^{13}C (100 MHz): δ 160.7 (C-2), 148.8 (C-7), 146.8 (C-2′), 144.5 (C-4), 144.0 (C-8a), 139.9 (C-3′′), 131.8 (C-8), 126.0 (C-6), 119.9 (C-2′′), 116.6 (C-4a), 114.8 (C-3), 113.3 (C-5), 106.8 (C-3′), 70.3 (C-1′′), 26.0 (C-4′′), 18.3 (C-5′′)

7. Compound **7**, Isoimperatorin, $CDCl_3$, ^{1}H (400 MHz): δ 8.15 (1H, d, *J* = 9.8 Hz, H-4), 7.59 (1H, d, *J* = 2.4 Hz, H-2′), 7.15 (1H, s, H-8), 6.95 (1H, dd, *J* = 2.4 Hz, H-3′), 6.27 (1H, d, *J* = 9.8 Hz, H-3), 5.54 (1H, tquint, *J* = 7.0 Hz, H-2′′), 4.92 (2H, d, *J* = 7.0 Hz, H-1′′), 1.80 (3H, s, H-5′′), 1.70 (3H, s, 4′′); ^{13}C (100 MHz): δ 161.5 (C-2), 158.3 (C-7), 152.8 (C-8a), 149.1 (C-5), 145.0 (C-2′), 140.0 (C-3′′), 139.7 (C-4), 119.2 (C-2′′), 114.4 (C-6), 112.7 (C-3), 107.7 (C-4a), 105.2 (C-3′), 94.4 (C-8), 69.9 (C-1′′), 26.0 (C-5′′), 18.4 (C-4′′)

8. Compound **8**, Phellopterin, $CDCl_3$, ^{1}H (400 MHz): δ 8.14 (1H, d, *J* = 9.8 Hz, H-4), 7.64 (1H, d, *J* = 2.3 Hz, H-2′) 7.01 (1H, d, *J* = 2.3 Hz, H-3′), 6.30 (1H, d, *J* = 9.8 Hz, H-3), 5.63 (1H, tq, *J* = 7.3 Hz, H-2′′), 4.87 (2H, d, *J* = 7.3 Hz, H-1′′), 4.20 (3H, s, O-CH_3), 1.76 (3H, s, H-4′′), 1.72 (3H, s, H-5′′); ^{13}C (100 MHz): δ 160.5 (C-2), 150.8 (C-5), 145.1 (C-8a), 145.1 (C-2′), 144.4 (C-7), 139.7 (C-3′′), 139.4 (C-4), 126.9 (C-8), 119.8 (C-2′′), 114.6 (C-6), 112.8 (C-3), 107.6 (C-4a), 105.0 (C-3′), 70.4 (C-1), 60.8 (O-CH_3), 25.8 (C-4′′), 18.1 (C-5′′)

9. Compound **9**, Oxypeucedanin hydrate, $CDCl_3$, ^{1}H (400 MHz): δ 8.17 (1H, d, *J* = 10.0 Hz, H-4), 7.61 (1H, d, *J* = 2.4 Hz, H-2′), 7.18 (1H, s, H-8), 6.99 (1H, dd, *J* = 2.4 Hz, H-3′), 6.29 (1H, d, *J* = 10 Hz, H-3), 4.54 (1H,

dd, *J* = 10 Hz, H_a-1′′), 4.44 (1H, dd, *J* = 10 and 7.8 Hz, H_b-1′′), 3.91 (1H, m, *J* = 7.8 Hz, H-2′′), 2.84 (1H, d, 2′′-OH), 2.16 (1H, s, 3′′-OH), 1.36 (3H, s, H-5′′), 1.32 (3H, s, H-4′′)); ^{13}C (100 MHz): δ 161.2 (C-2), 158.2 (C-7), 152.7 (C-8a), 148.6 (C-5), 145.4 (C-2′), 139.1 (C-4), 114.4 (C-6), 113.3 (C-3), 107.5 (C-4a), 104.8 (C-3′), 95.1 (C-8), 76.6 (C-2′′), 74.6 (C-1′′), 71.8 (C-3′′), 26.9 (C-5′′), 25.3 (C-4′′)

10. Compound **10**, Oxypeucedanin methanolate, $CDCl_3$, ^{1}H (400 MHz): δ 8.23 (1H, dd, *J* = 10.0 Hz, H-4), 7.60 (1H, d, *J* = 2.4 Hz, H-2′), 7.17 (1H, bs, H-8), 7.00 (1H, dd, *J* = 2.4 Hz, H-3′), 6.29 (1H, d, *J* = 10.0 Hz, H-3), 4.57 (1H, dd, *J* = 10 and 7.8 Hz, H_a-1′′), 4.39 (1H, dd, *J* = 10 Hz, H_b-1′′), 3.94 (1H, m, *J* = 7.8 Hz, H-2′′), 3.27 (3H, s, 3′′-O-CH_3), 2.68 (1H, d, 2′′-OH), 1.27 (3H, s, H-5′′), 1.24 (3H, s, H-4′′); ^{13}C (100 MHz): δ 161.3 (C-2), 158.3 (C-7), 152.8 (C-8a), 149.0 (C-5), 145.2 (C-2′), 139.5 (C-4), 114.2 (C-2), 113.1 (C-3), 107.5 (C-4a), 105.0 (C-3′), 94.7 (C-8), 76.3 (C-2′′), 76.1 (C-3′′), 74.4 (C-1′′), 49.4 (3′′-O-CH_3), 20.9 (C-5′′), 20.9 (C-4′′)

11. Compound **11**, Ostruthol, $CDCl_3$, ^{1}H (400 MHz): δ 8.08 (1H, dd, *J* = 9.8 Hz, H-4), 7.60 (1H, d, *J* = 2.4, H-2′), 7.15 (1H, bs, H-8), 6.97 (1H, dd, *J* = 2.4 Hz, H-3′), 6.25 (1H, d, *J* = 9.8 Hz, H-3), 6.14 (1H, qq, *J* = 7.4 Hz, H-3′′′), 5.40 (1H, dd, *J* = 8 Hz, H-2′′), 4.83 (1H, dd, *J* = 10.0 Hz, H_a-1′′), 4.64 (1H, dd, *J* = 10.0 and 8.0 Hz, H_b-1′′), 1.99 (3H, dq, *J* = 7.4 Hz, H-4′′′), 1.88 (3H, quint, H-5′′′), 1.37 (3H, s, H-4′′), 1.34 (3H, s, H-5′′); ^{13}C (100 MHz): δ 167.3 (C-1′′′), 161.3 (C-2), 158.3 (C-7), 152.8 (C-8a), 148.7 (C-5), 145.2 (C-2′), 139.8 (C-3′′′), 139.4 (C-4), 127.3 (C-2′′′), 113.4 (C-6), 113.0 (C3), 107.0 (C-4a), 94.5 (C-8), 77.2 (C-2′′), 71.8 (C-1′′), 26.8 (C4′′), 26.6 (C-5′′), 20.7 (C-5′′′), 16.0 (C-4′′′)

12. Compound **12**, Oxypeucedanin, $CDCl_3$, ^{1}H (400 MHz): δ 8.21 (1H, dd, *J* = 9.8 Hz, H-4), 7.61 (1H, d, *J* = 2.4 Hz, H-2′), 7.20 (1H, bs, H-8), 6.95 (1H, dd, *J* = 2.4 Hz, H-3′), 6.32 (1H, d, *J* = 9.8 Hz, H-3), 4.60 (1H, dd, *J* = 10.8 and 4.4 Hz, H_a-1′′), 4.44 (1H, dd, *J* = 10.8 and 6.6 Hz, H_b-1′′), 3.23 (1H, dd, *J* = 6.6 and 4.4 Hz, H-2′′), 1.41 (3H, s, H-5′′), 1.33 (3H, s, H-4′′); ^{13}C (100 MHz): δ 161.2 (C-2), 158.2 (C-7), 152.7 (C-8a), 148.5 (C-5), 145.5 (C-2′), 139.1 (C-4), 114.4 (C-6), 113.4 (C-3), 107.6 (C-4a), 104.6 (C-3′), 95.1 (C-8), 72.5 (C-1′′), 61.3 (C-2′′), 58.5 (C-3′′), 24.8 (C-5′′), 19.2 (C-4′′)

13. ^{1}H NMR spectrum of compound **11**

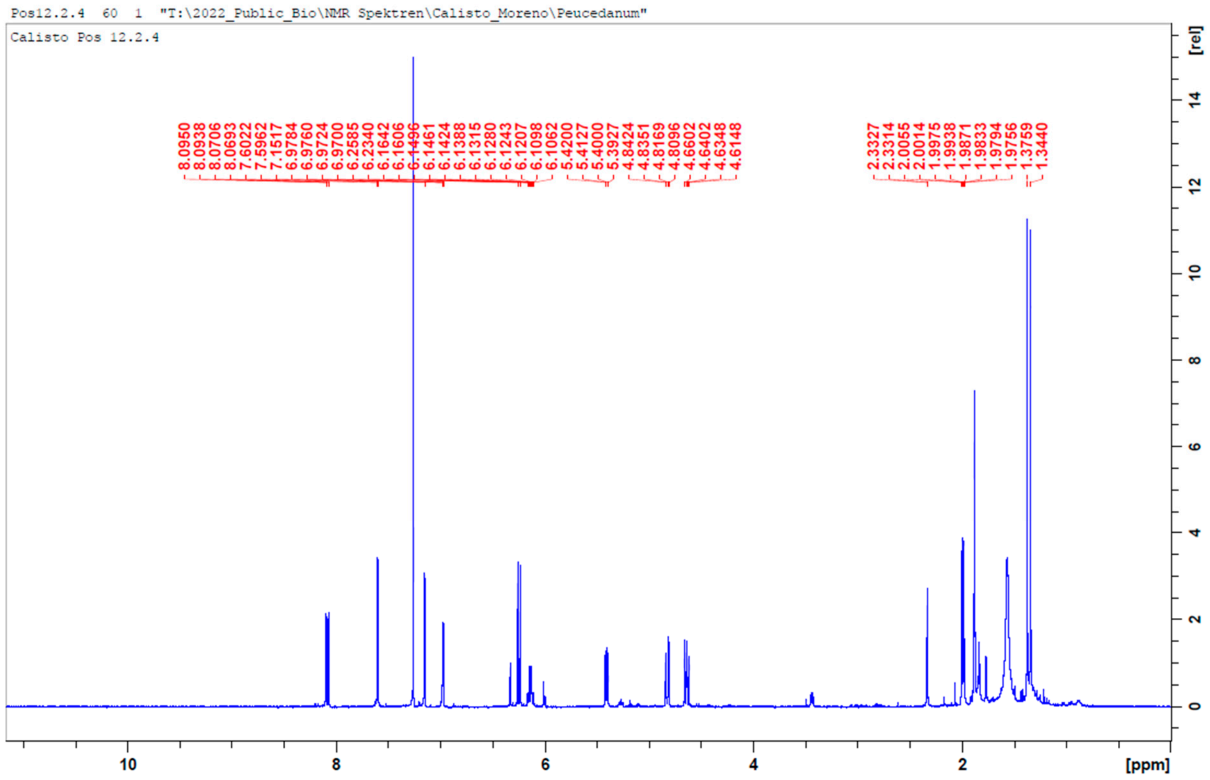

14. ^{13}C NMR spectrum of compound **11**

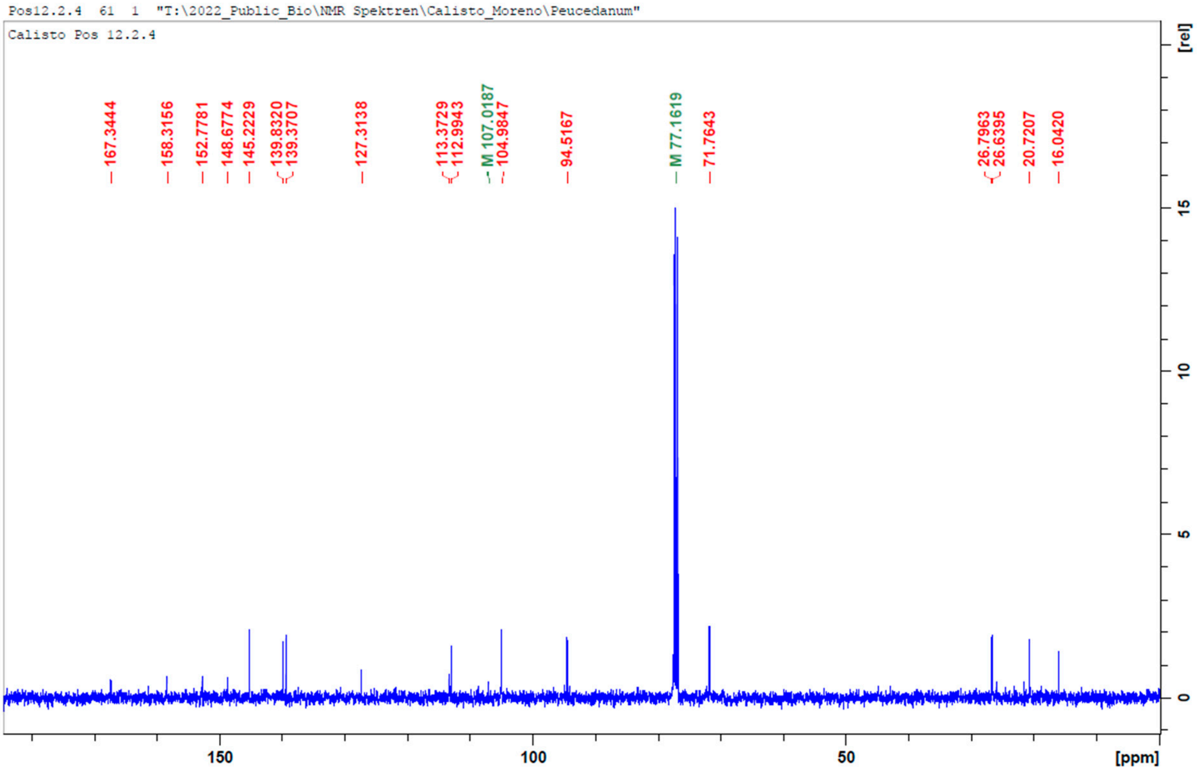
Pos12.2.4 61 1 "T:\2022_Public_Bio\NMR Spektren\Calisto_Moreno\Peucedanum"
Calisto Pos 12.2.4
167.3444
158.3156
152.7781
148.6774
145.2229
139.8320
139.3707
127.3138
113.3729
112.9943
M 107.0187
104.9847
94.5167
M 77.1619
71.7643
26.7963
26.6395
20.7207
16.0420
[rel]
[ppm]

15. COSY spectrum of compound **11**

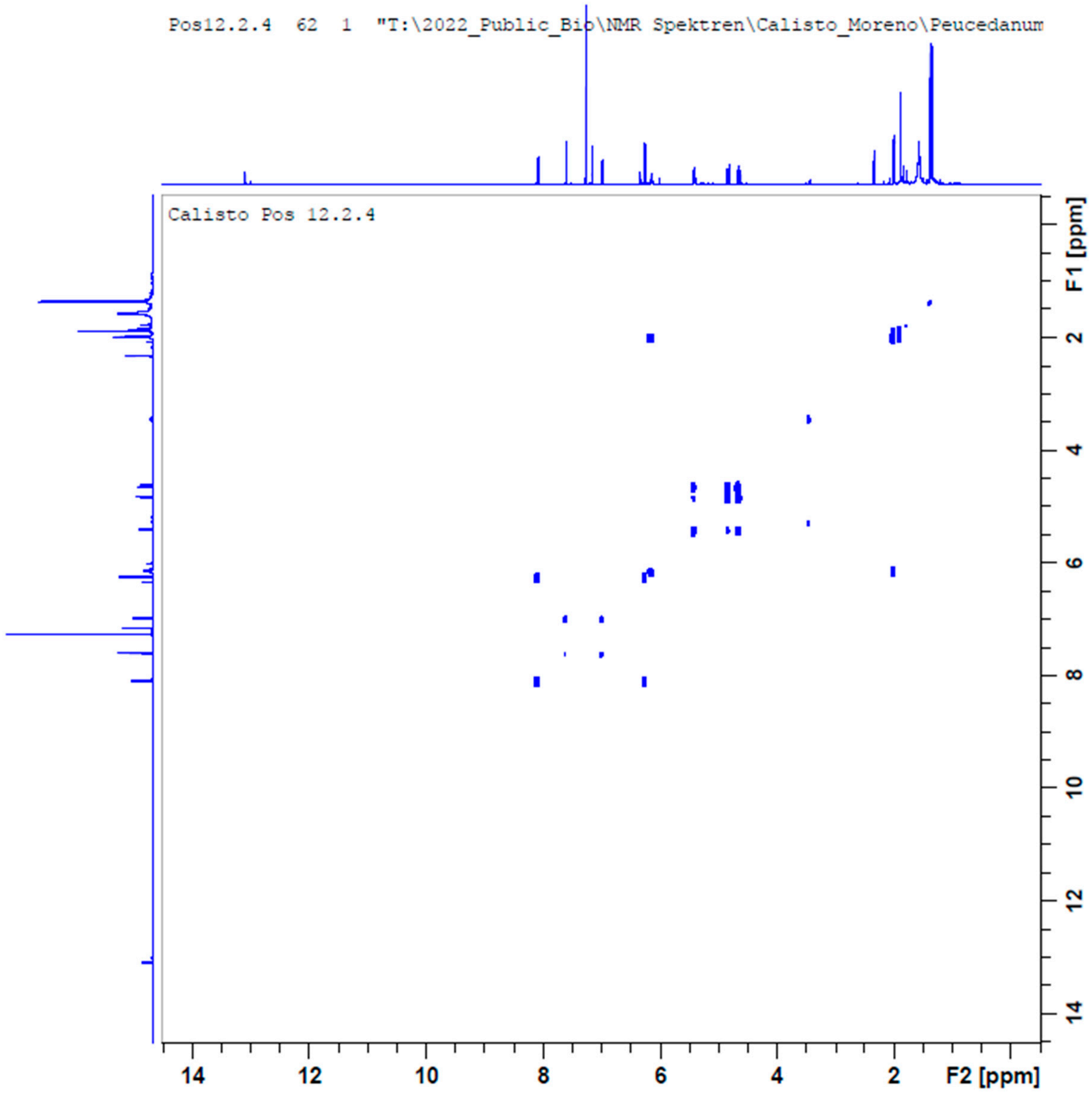

16. HSQC spectrum of compound **11**

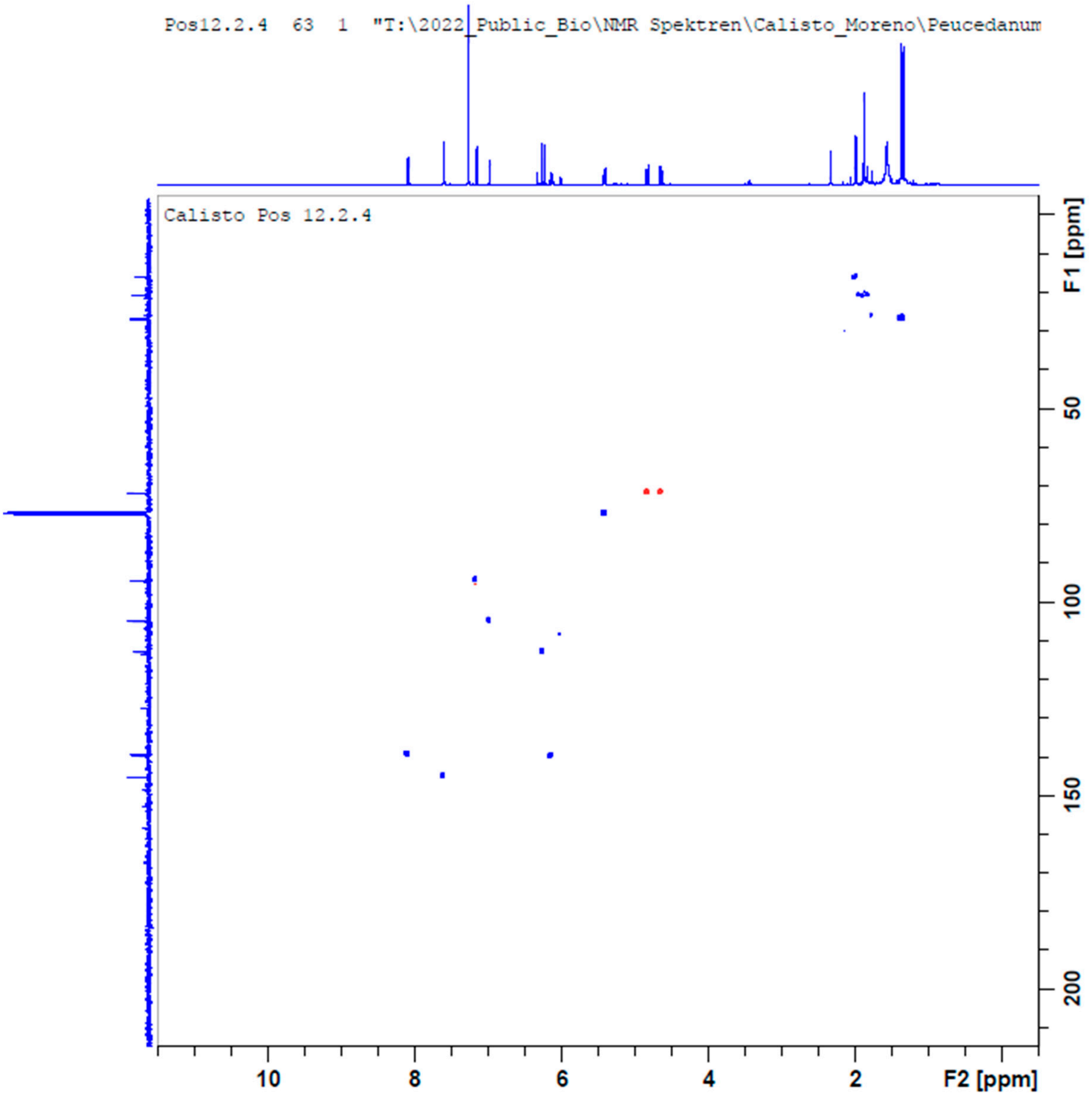

17. HMBC spectrum of compound **11**

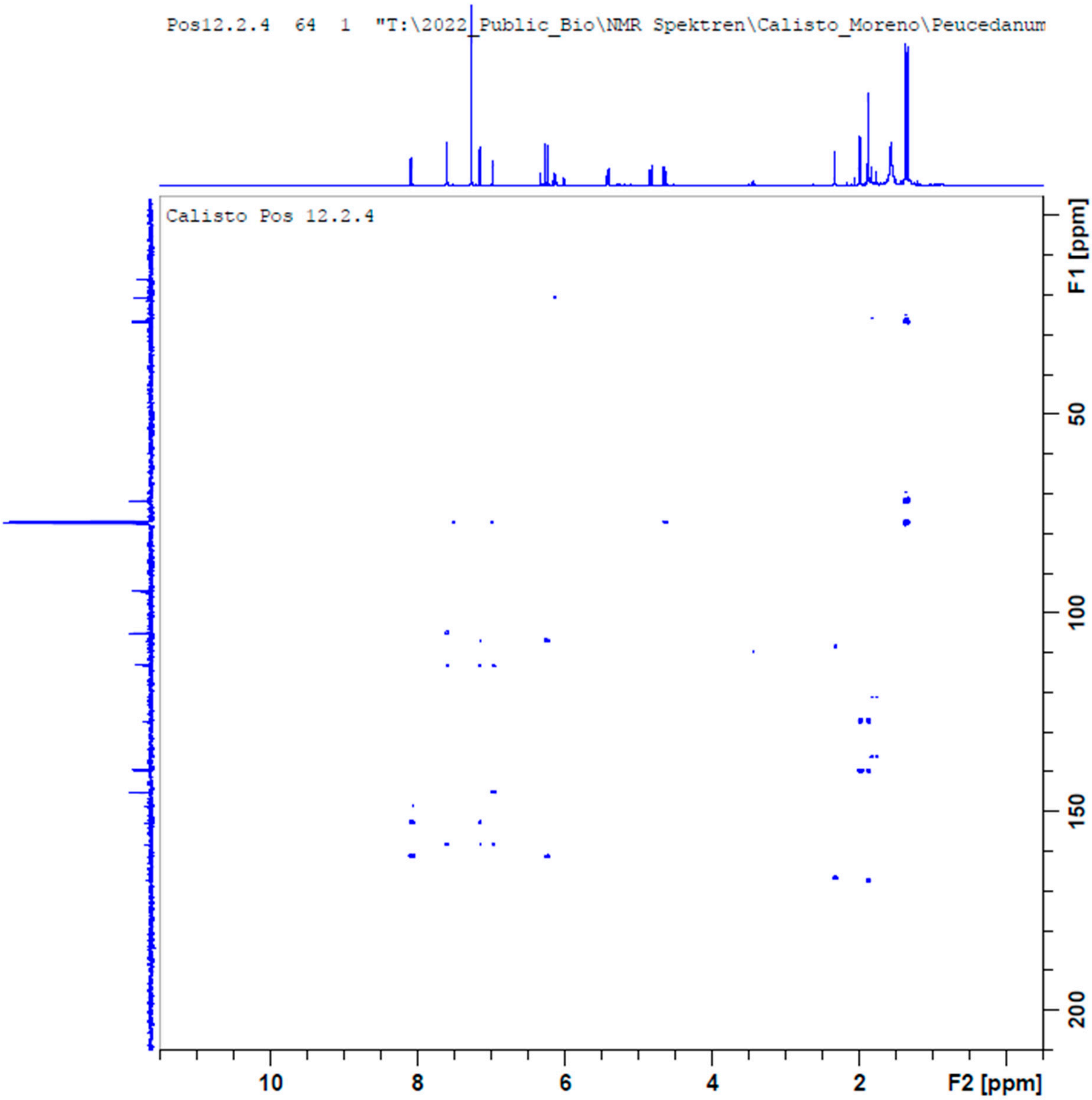

18. MS and MS/MS spectra of Compound **1**

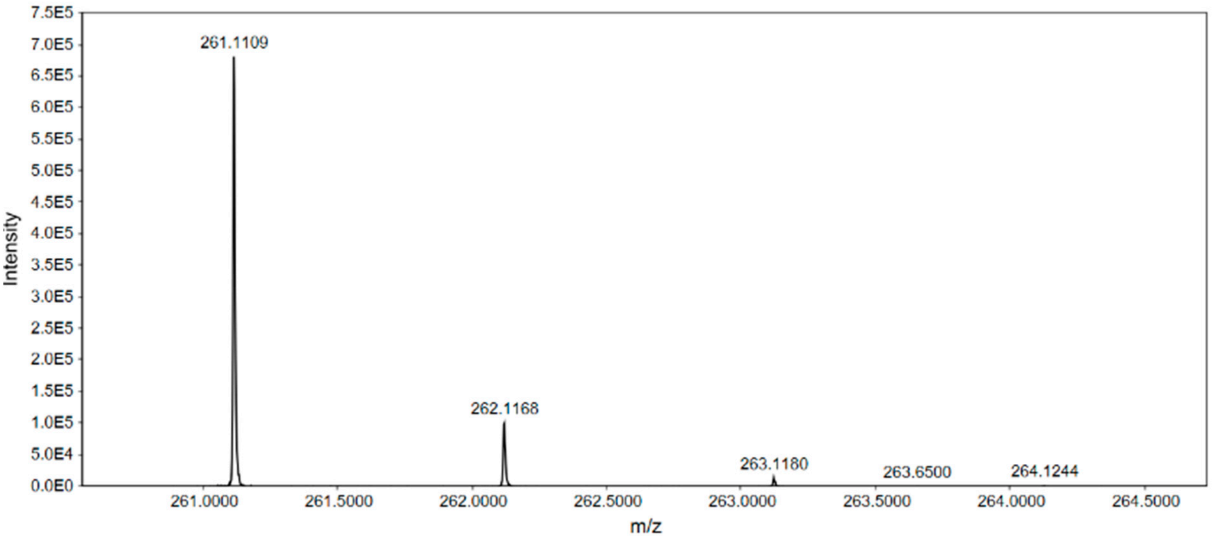

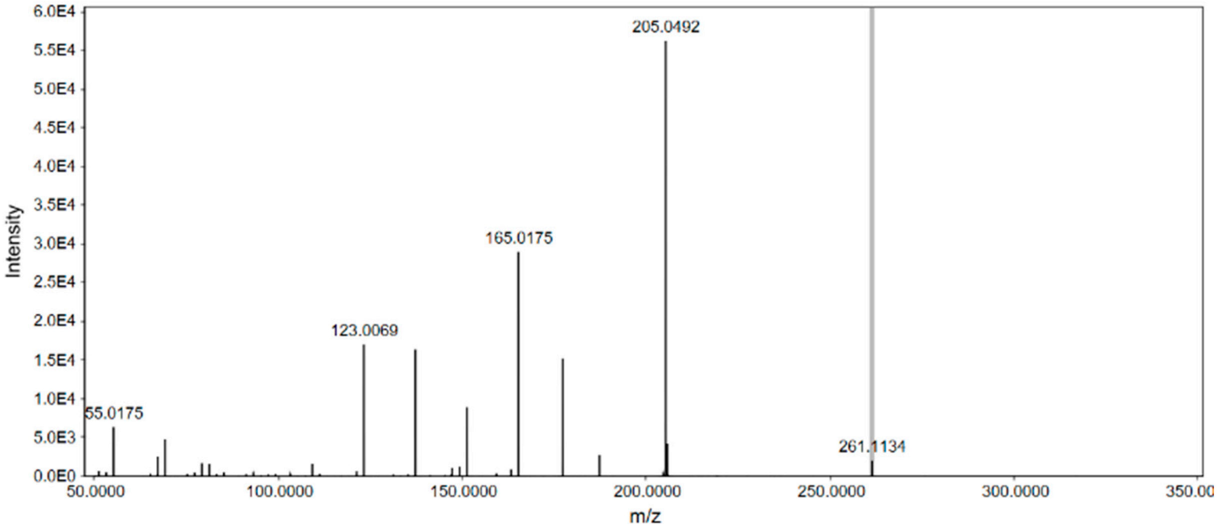

19. MS and MS/MS spectra of compound **2**

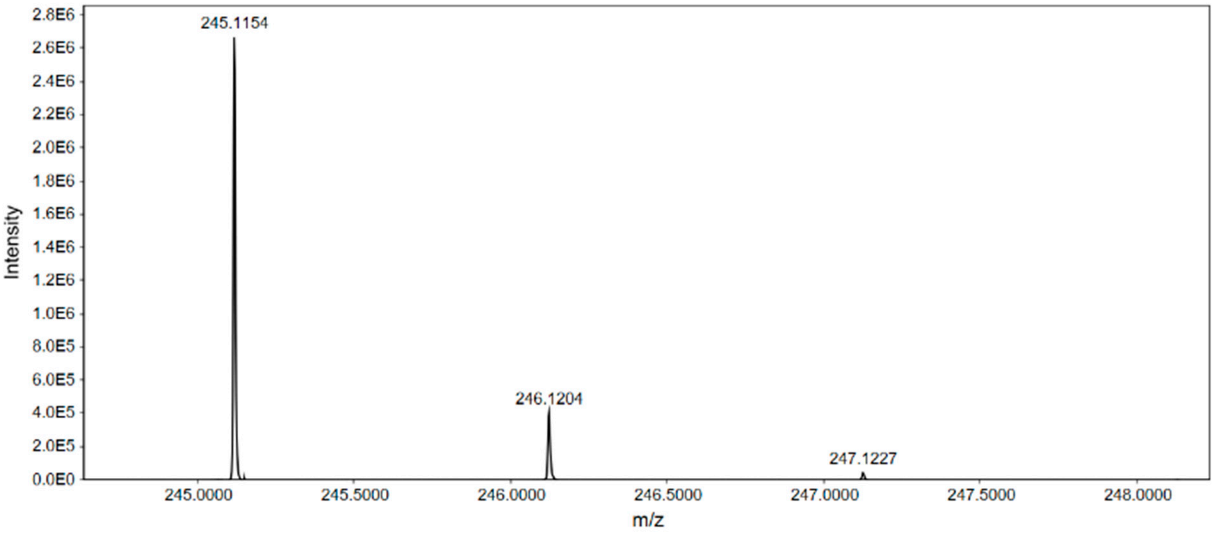

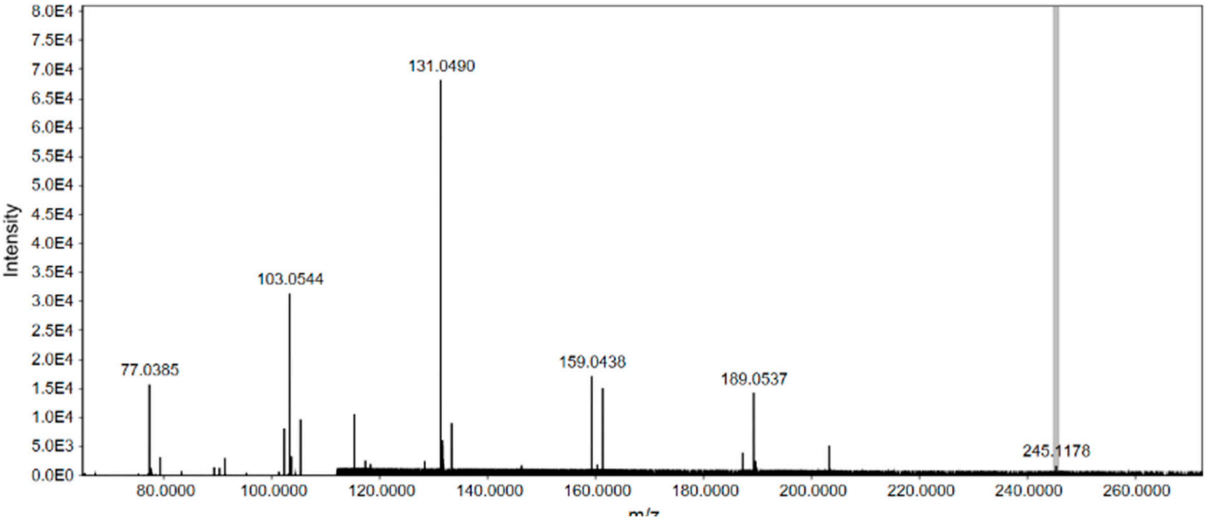

20. MS and MS/MS spectra of compound **3**

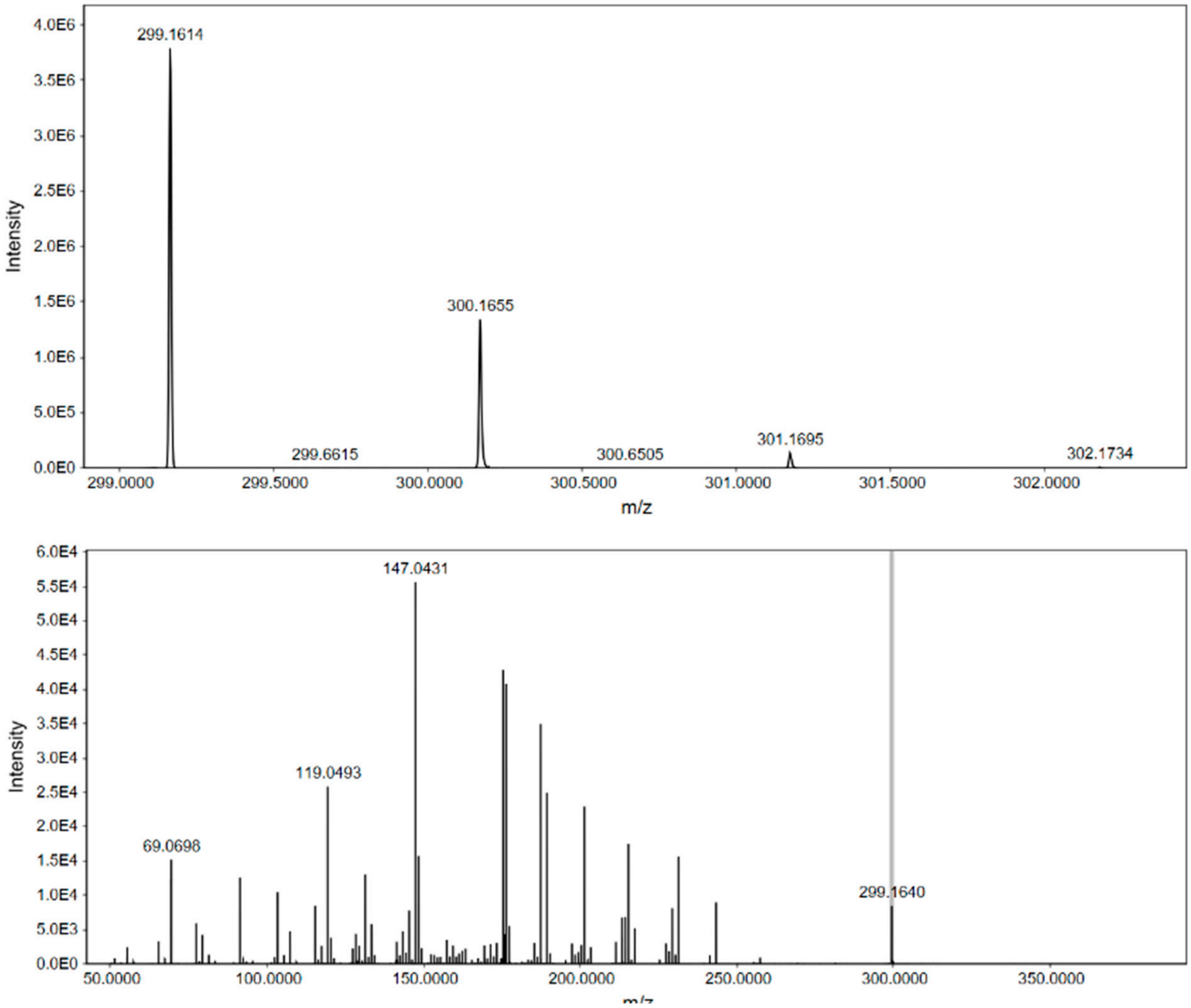

21. MS and MS/MS spectra of compound **4**

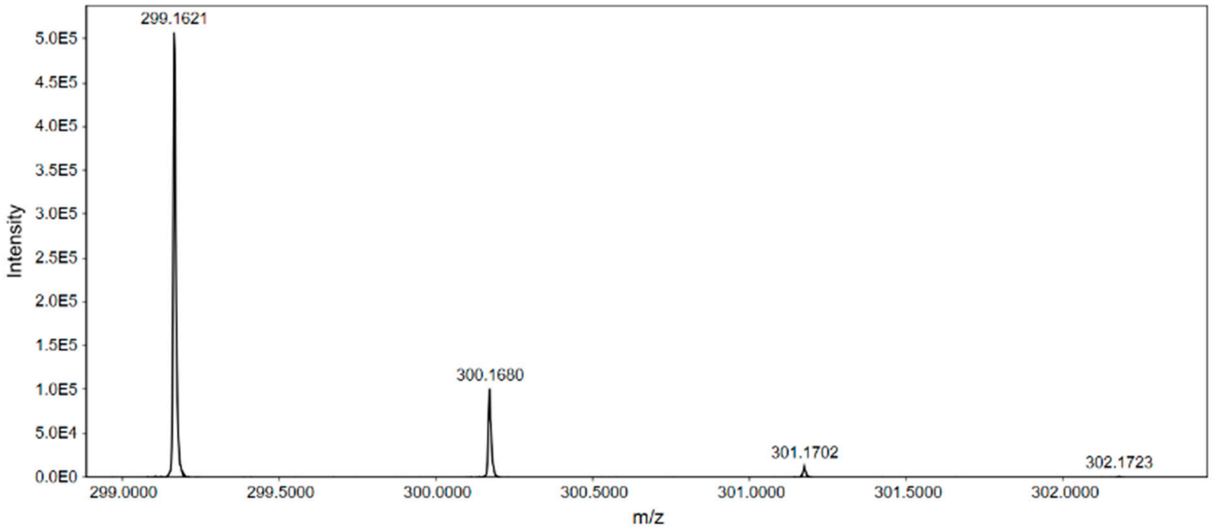

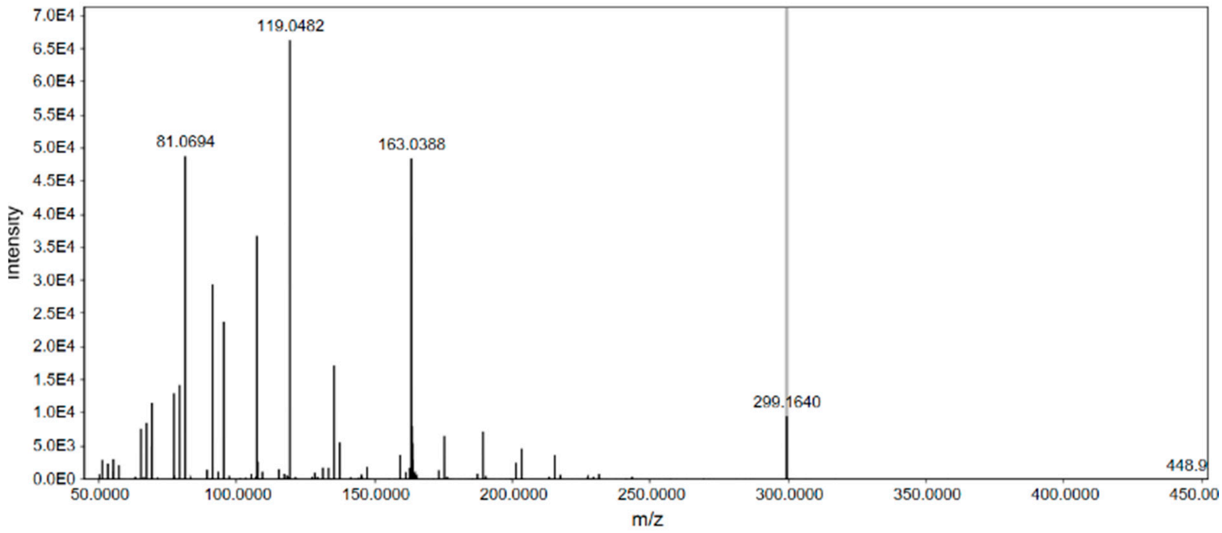

22. MS and MS/MS spectra of compound **5**

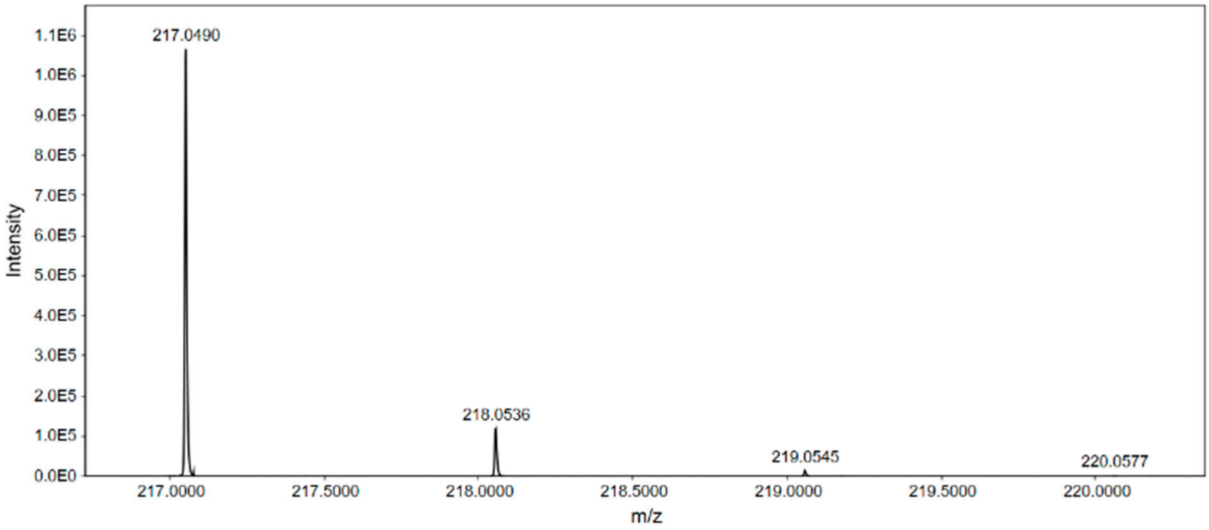

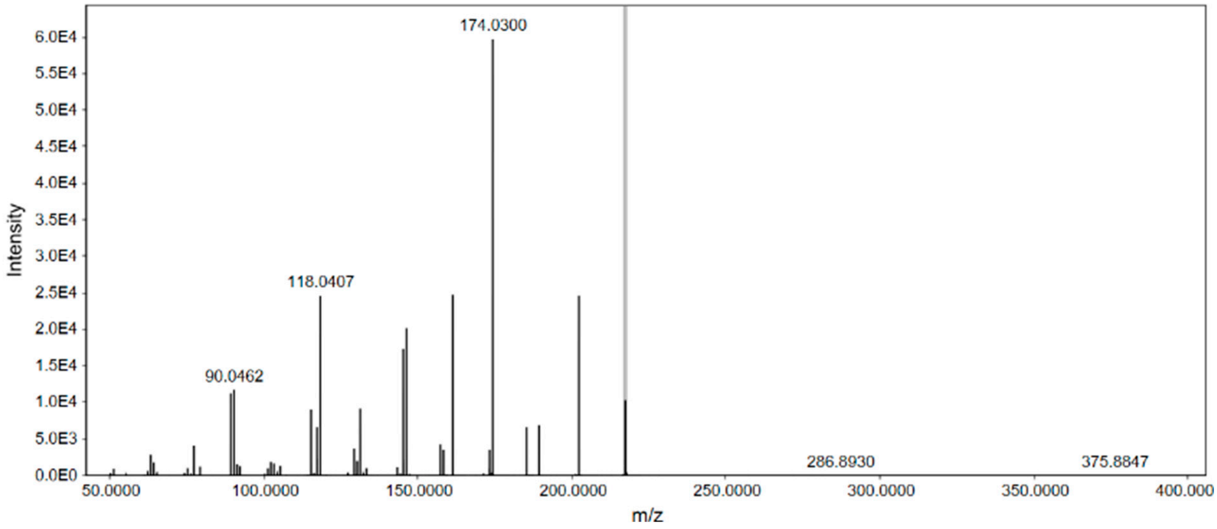

23. MS and MS/MS spectra of compound **6**

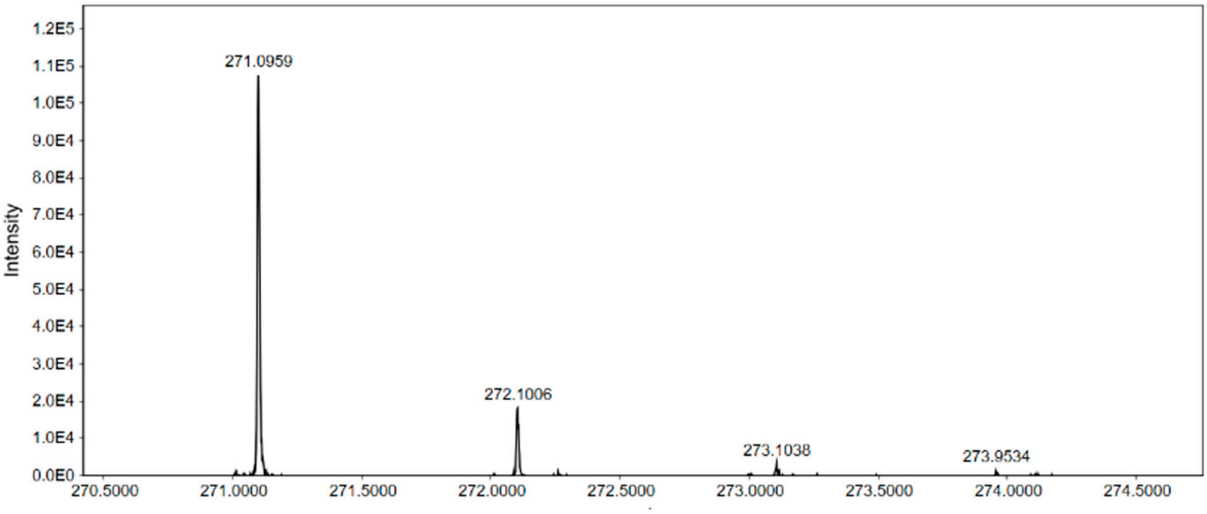

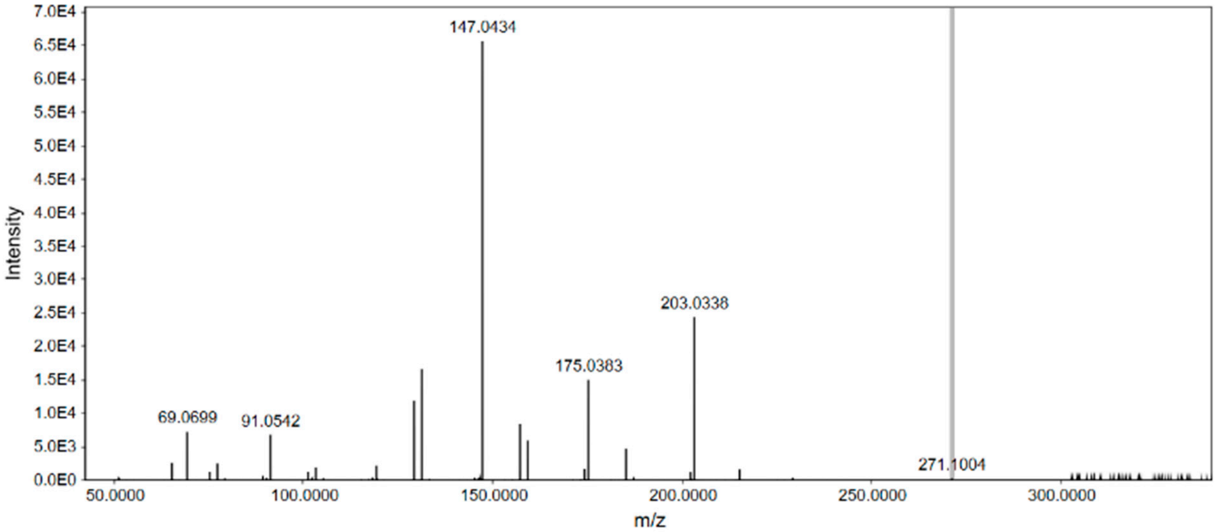

24. MS and MS/MS spectra of compound **7**

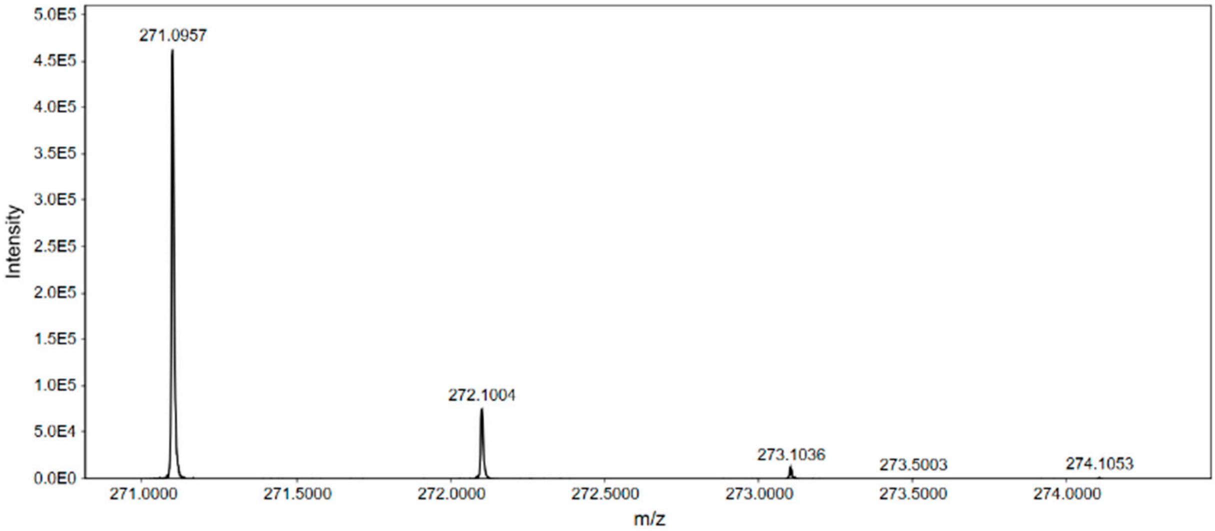

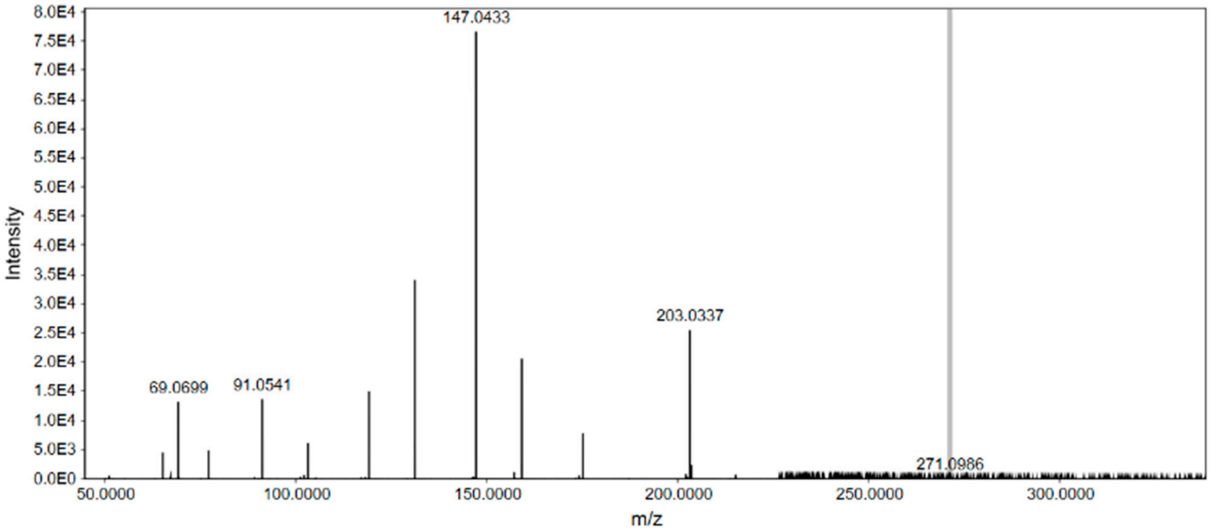

25. MS and MS/MS spectra of compound **8**

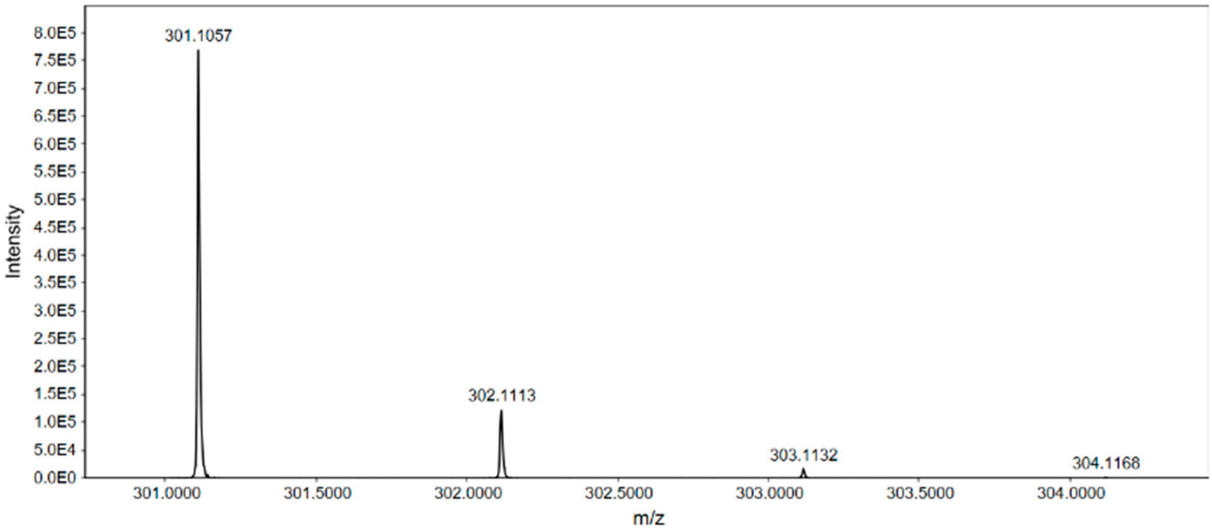

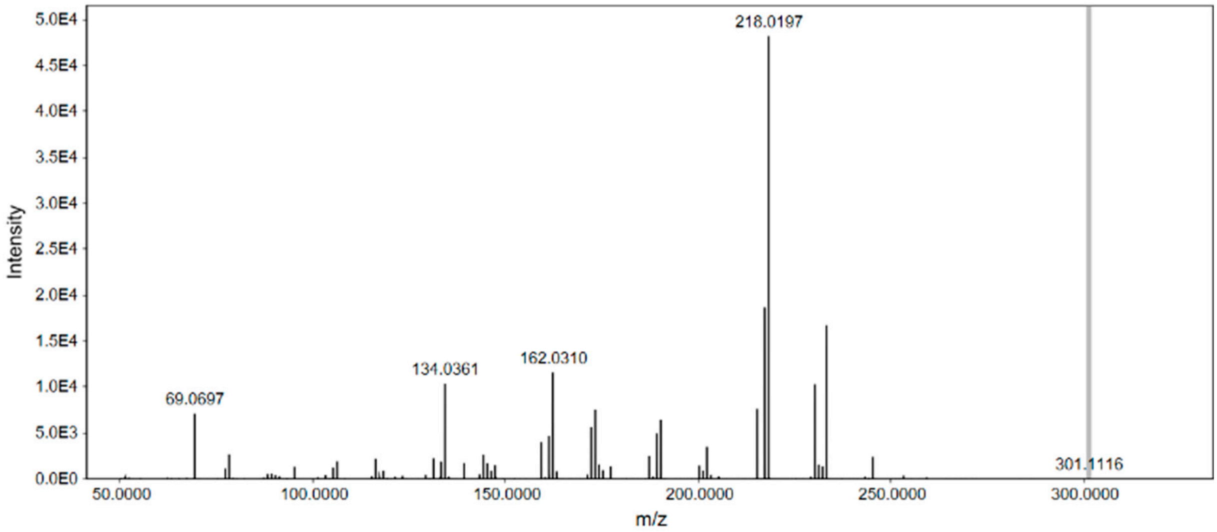

26. MS and MS/MS spectra of compound **9**

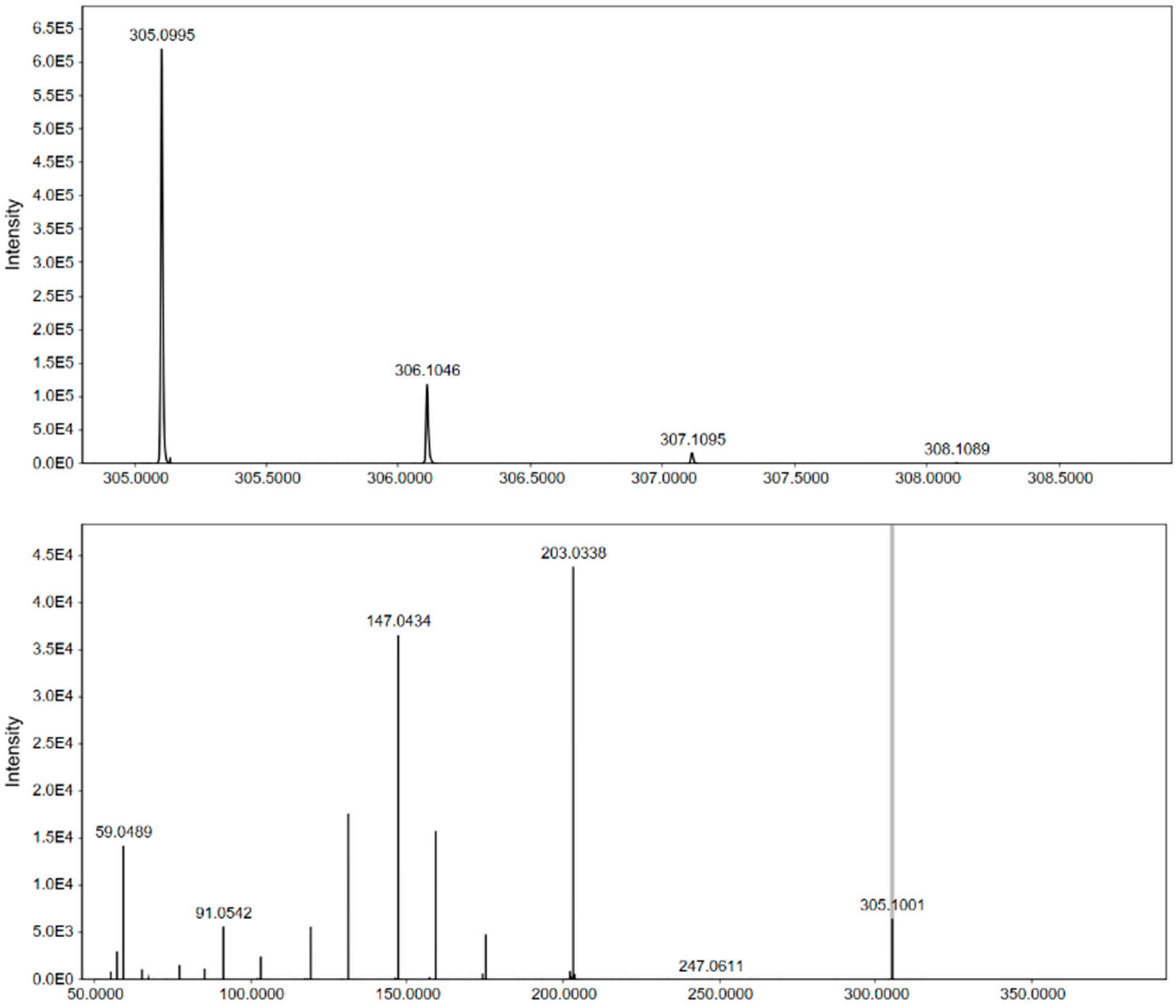

27. MS and MS/MS spectra of compound **10**

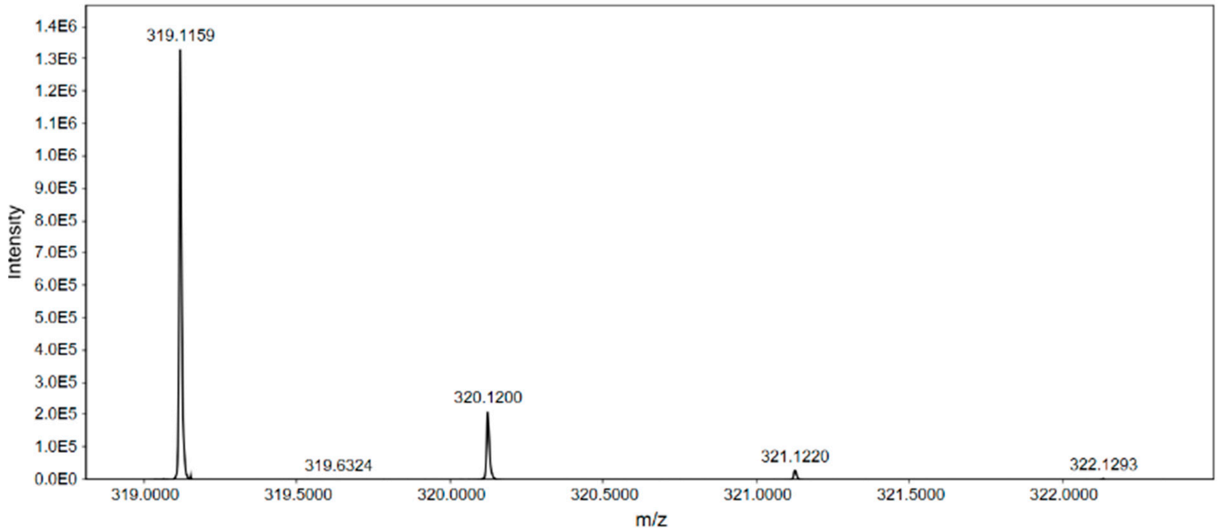

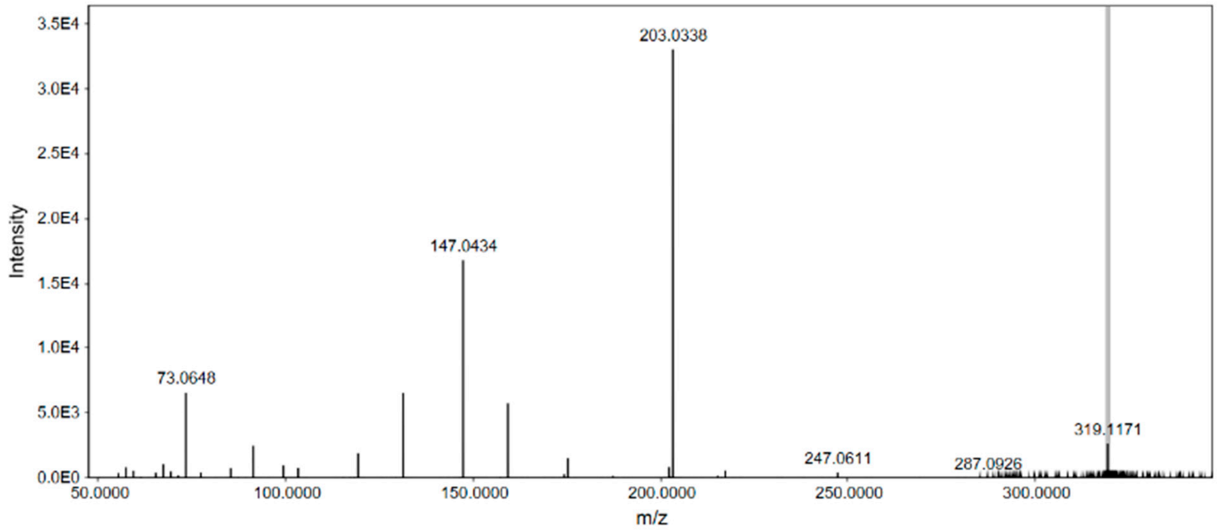

28. MS and MS/MS spectra of compound **11**

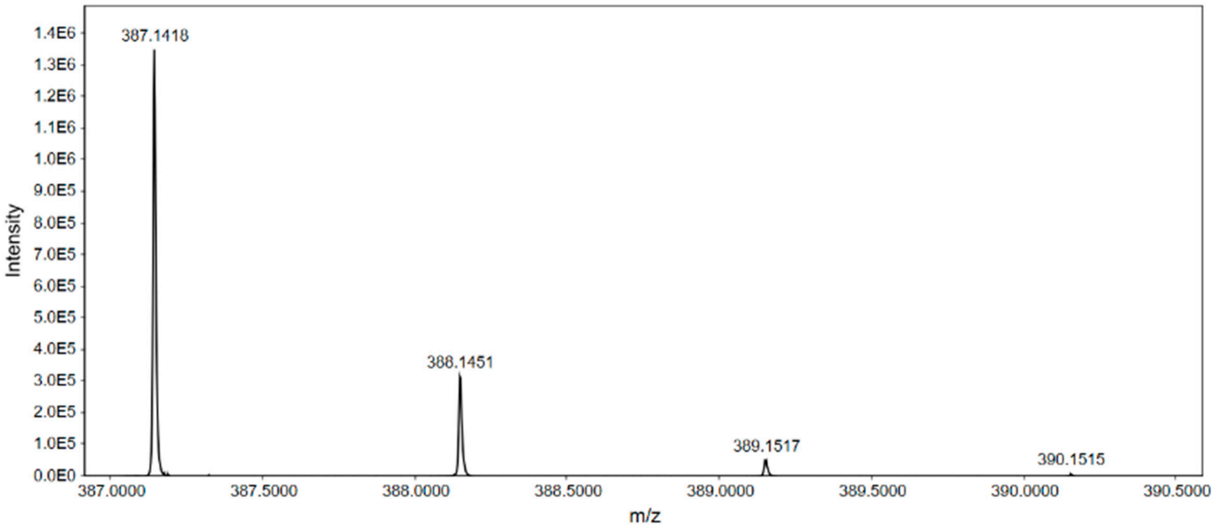

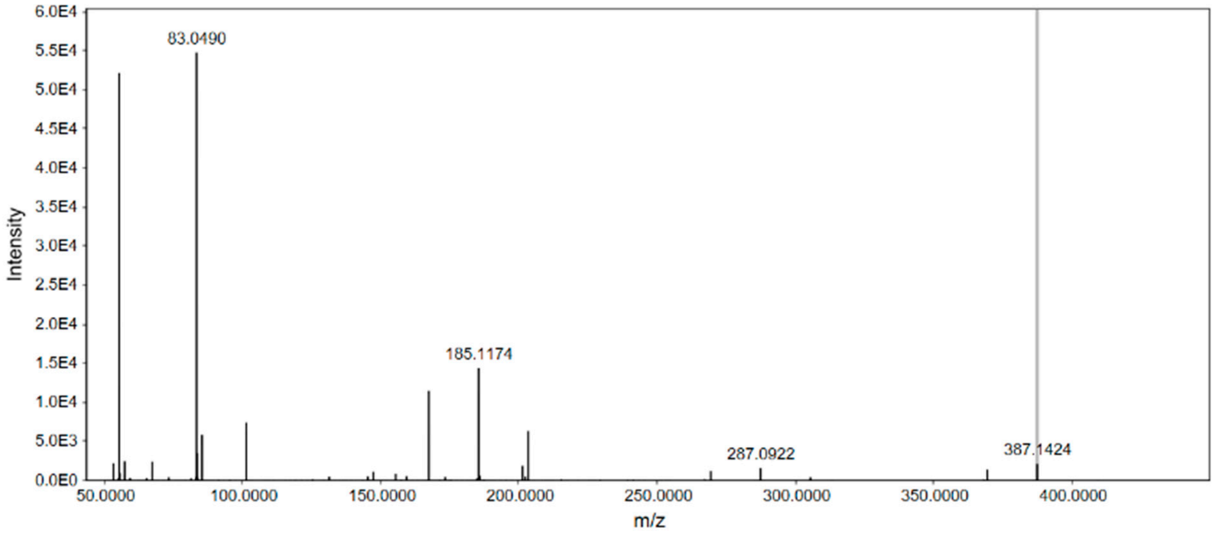

29. MS and MS/MS spectra of compound **12**

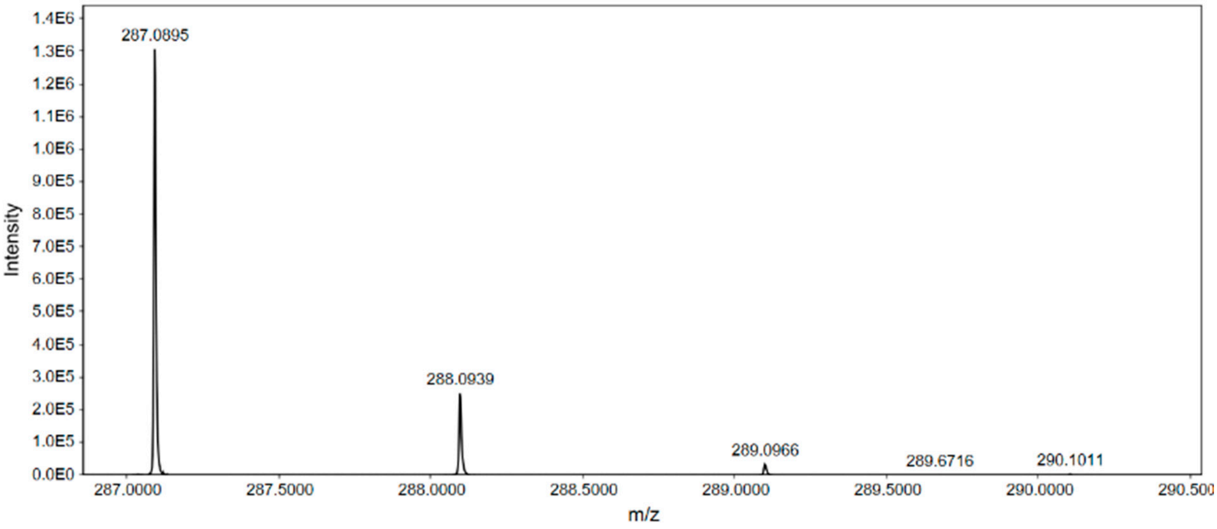

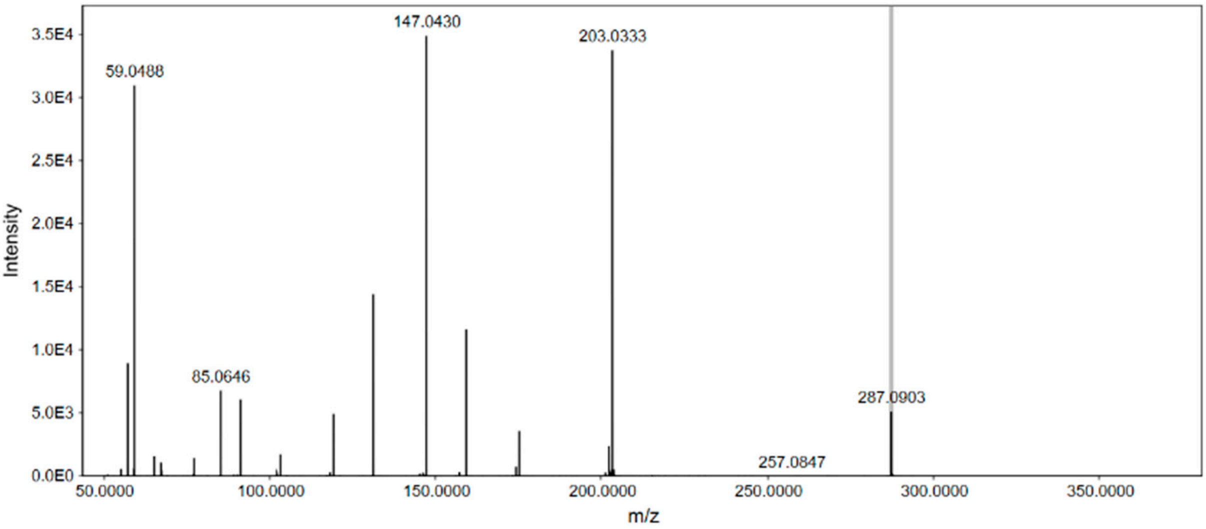

Supplement: Supplementary file 1 [file plants-14-02815-s001.zip › plants-3787912-supplementary.pdf]
